# Diagnostic accuracy of tests for assessing readiness for liberation from mechanical ventilation in adults: an overview of reviews

**DOI:** 10.1186/s40560-026-00848-9

**Published:** 2026-01-14

**Authors:** Carlos Fernando Grillo-Ardila, Luis Carlos Triana-Moreno, Carlos Eduardo Laverde-Sabogal, Javier Andrés Mora-Arteaga, Miguel Angel Aguilar-Schotborgh, Juan José Ramírez-Mosquera

**Affiliations:** 1 School of Medicine, Universidad Nacional de Colombia, 111321 Bogotá, Colombia; 2 Intensive Care Unit, Clínica del Country, 110231 Bogotá, Colombia; 3 Department of Internal Medicine, School of Medicine, Pontificia Universidad Javeriana, 110231 Bogotá, Colombia; 4 Intensive Care Unit, Hospital Universitario San Ignacio, 110231 Bogotá, Colombia; 5 School of Medicine, Pontificia Universidad Javeriana, 110231 Bogotá, Colombia

**Keywords:** Ventilator weaning, Airway extubation, Respiratory insufficiency, Ventilators mechanical, Diagnostic test routine

## Abstract

**Objectives:**

To summarize the evidence on the accuracy of tests evaluating readiness for liberation from mechanical ventilation in the adult population.

**Materials and methods:**

Searches were conducted in MEDLINE, Embase, CENTRAL, and CINAHL, with additional publications identified through conference proceedings and contact with experts. Systematic reviews (SRs) were independently assessed for inclusion, data extraction, and risk of bias, without language or date restrictions. Included SRs focused on adults diagnosed with ventilatory failure requiring invasive support for more than 24 h. Successful weaning was considered being alive in absence of ventilatory support 72 h following liberation from mechanical ventilation.

**Results:**

Ten SRs examining the diagnostic accuracy of 23 readiness tests were included. These tests were conducted before, during, or after spontaneous breathing trials using various methods, such as pressure support, T-piece and continuous positive airway pressure. Among these, lung ultrasound score (sensitivity 0.94, 95% CI 0.59–0.99; specificity 0.87, 95% CI 0.62–0.97), diaphragmatic rapid shallow breathing index (sensitivity 0.84, 95% CI 0.76–0.90; specificity 0.87, 95% CI 0.79–0.92), venous oxygen saturation (sensitivity 0.83, 95% CI 0.74–0.90; specificity 0.88, 95% CI 0.83–0.92), and brain natriuretic peptide (sensitivity 0.88, 95% CI 0.83–0.92; specificity 0.82, 95% CI 0.73–0.89), showed high to moderate diagnostic capacity for ruling in and ruling out weaning failure. The remaining tests (e.g., cuff leak test, cough peak flow, P0.1, RSBI, MIP, DE, DTF, DTF–RSBI, EeDT, and EiDT) demonstrated weak diagnostic accuracy. In the SRs, risk of bias ranged from low to high.

**Conclusions:**

The accuracy of tests used to assess readiness for withdrawing ventilatory support in adults varies considerably. Physicians should integrate the results of physiological, ultrasound, and paraclinical measures to minimize uncertainty in deciding which patients should progress toward ventilator weaning, always considering individual patient needs to ensure personalized healthcare.

**Supplementary Information:**

The online version contains supplementary material available at 10.1186/s40560-026-00848-9.

## Introduction

Liberation from mechanical ventilation represents a continuous process that begins as soon as the decision to initiate mechanical support is made [1, 2], and involves a series of crucial considerations, such as the timely recognition of a reasonable probability of successful extubation, the assessment of readiness parameters, the spontaneous breathing test and the removal of the endotracheal tube [2]. However, the removal of this support is not without risks. Weaning failure (e.g., the need to restart ventilatory support in the first 72 h after the scheduled removal of the endotracheal tube) affects 4–25% of patients, and has a negative impact on health outcomes [3, 4], as it increases mortality and the incidence of aspiration, atelectasis, and pneumonia while prolonging hospital stay, the need for tracheostomy and healthcare costs (US$33,926) [5].

On the other hand, delay in weaning from mechanical ventilation also has a deleterious effect on critically ill patients, as it exposes them to the development of delirium [6], diaphragmatic atrophy, physical deconditioning, and ventilator-associated pneumonia, adversely impacting their stay in the ICU [7]. Patients with prolonged mechanical ventilation represent 6% of all ventilated patients but consume 37% of the resources in the ICU. This explains why the optimal time for weaning represents one of the most challenging issues in the care of critically ill patients. It is imperative to identify those patients who have a greater probability of being successfully weaned from mechanical ventilation, and carefully select the right timing [8]. Timing should be established based on the evaluation of a series of biological and functional parameters that allow confirmation of clinical suspicion, reducing the level of uncertainty to an acceptable point, where the performance of a spontaneous breathing test with subsequent removal of the tube is justified [9].

Given the critical importance of this topic in intensive care, a significant body of literature has emerged on the diagnostic accuracy of readiness tests for weaning patients from mechanical ventilation. However, these studies often yield inconsistent results. Systematic reviews (SRs), with or without meta-analyses, are vital for decision-making, as they consolidate the best available evidence. Nonetheless, accepting their findings without a critical evaluation poses risks; methodological flaws can lead to erroneous conclusions. Thus, this overview of systematic reviews [10] aims to identify, analyze and synthesize the most reliable evidence regarding the accuracy of tests for assessing readiness for liberation from mechanical ventilation in adults, ultimately reducing uncertainty in clinical decision-making.

## Materials and methods

The protocol was designed in accordance with the recommendations of the Cochrane Handbook for Systematic Reviews [10] and the Cochrane Handbook for Systematic Reviews of Diagnostic Test Accuracy [11], and registered in PROSPERO (CRD42024479426; available at: https://www.crd.york.ac.uk/prospero/display_record.php?ID=CRD42024479426). Ethical approval was obtained from the ethics committee of Pontificia Universidad Javeriana. The results were reported as per the PRISMA–DTA statement [12].

### Literature search

A structured search using a combination of controlled vocabulary and free-text terms was conducted in MEDLINE, Embase, CENTRAL, and CINAHL from inception until November 10, 2025. To identify additional relevant reviews, a search was conducted in the gray literature, from conference proceedings of the Society of Critical Care Medicine (SCCM) and the European Society of Intensive Care Medicine (ESICM). The referenced lists of all included studies and expert content were contacted to identify additional publications. There were no language or date restrictions (Additional file 1).

### Eligibility and exclusion criteria

The selection criteria for this overview of reviews were SRs that assessed the accuracy of pulmonary function tests (e.g., airway occlusion pressure at 100 ms, rapid shallow breathing index, maximal inspiratory pressure, cuff leak test, and cough peak flow), ultrasound-guided pulmonary and diaphragmatic evaluation (e.g., lung ultrasound score, diaphragmatic excursion, diaphragmatic rapid shallow breathing index, diaphragmatic thickening fraction, end-expiratory diaphragm thickness, and end-inspiratory diaphragm thickness), cardiovascular function tests (e.g., venous oxygen saturation and brain natriuretic peptide), or any other test to determine readiness for weaning from mechanical ventilation. Included reviews involved adult population diagnosed with ventilatory failure requiring invasive ventilatory support for more than 24 h in a hospital setting (e.g., intensive care unit and emergency department), regardless of the underlying pathology (e.g., medical, surgical, traumatic, or neurological) or the type of ventilatory failure (e.g., hypoxemic or hypercapnic). Successful weaning was considered being alive in absence of ventilatory support 72 h following liberation from mechanical ventilation.

### Data extraction

Two authors independently screened titles and abstracts, conducted the review selection and extracted relevant information using a data collection form designed in accordance with the Cochrane guidelines [10, 11]. Extracted data encompassed review questions, study eligibility criteria, identification and selection of studies, number of included studies, data collection methods, study appraisal, synthesis of findings, assessment of heterogeneity, and evaluation of publication bias. In addition, variables such as population characteristics, clinical settings, details of the index test, including cutoffs, and the reference standard were also recorded.

### Assessment of methodological quality

The quality of included reviews was evaluated using the Risk of Bias in Systematic Reviews (ROBIS) tool [13]. ROBIS is an instrument designed to assess the risk of bias in systematic reviews. It consists of three appraisal phases that examine relevance and flaws during the evaluation process (e.g., eligibility criteria, identification and selection of studies, data collection, and study appraisal, synthesis, and findings), ending with a judgment about the overall risk of bias in the interpretation of review findings [13]. Discrepancies were resolved through consensus and, if necessary, by consultation with a third author.

In cases where multiple SRs assessed the diagnostic accuracy of the same index test in the same population, priority was given including the highest quality meta-analysis based on the results of the ROBIS assessment, thus avoiding duplication of studies within this review. If SRs received the same ROBIS rating, preference was given to the most recently published study with the largest number of primary studies, prioritizing the most comprehensive review. The degree of publication overlap in this umbrella review was quantified using the corrected covered area (CCA) index. For this purpose, a citation matrix was constructed, listing all primary publications and the different systematic reviews included herein in rows and columns, respectively [14].

### Data analysis

For each selected SR, diagnostic test accuracy was extracted, including sensitivity, specificity, positive likelihood ratio, negative likelihood ratio, diagnostic odds ratio (DOR), and area under the curve (AUC) [15]. When a meta-analysis was not performed, but sufficient data were available to construct a 2 × 2 table for each included study, sensitivity and specificity (along with their 95% confidence intervals) were estimated, and a meta-analysis of diagnostic accuracy was conducted using the hierarchical summary receiver operating characteristic (HSROC) model. This approach was chosen, because this model takes into account within-study and between-study variation and focuses on estimating a summary operating point, including aggregate values for sensitivity and specificity, as well as the 95% confidence and prediction regions around this summary point. All statistical analyses were conducted using the STATA 18 software package. The overall quality of evidence was graded using the GRADE approach. The GRADE methodology evaluates the strength of a body of evidence on diagnostic tests according to risk of bias, consistency of effect, imprecision, indirectness, and publication bias criteria [16].

## Results

**Fig. 1:**
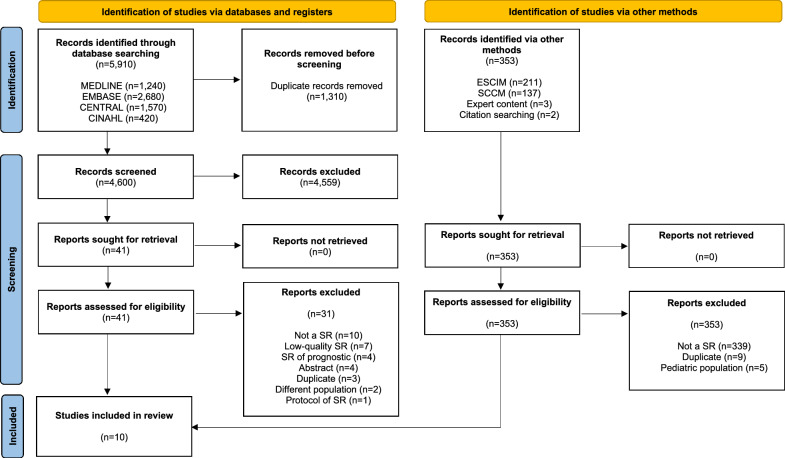
Study flowchart. Inclusion of studies at different stages for this systematic review and meta-analysis. From [42]

The search yielded a total of 6,263 references (Fig. 1). After eliminating duplicates, 4,953 references were screened. Out of these, 41 were reviewed in full text. Of those, 31 references were excluded due to reasons, such as not relevant design (*n* = 15), High risk of bias identified based on the ROBIS assessment (*n* = 7), abstracts (*n* = 4), duplicates (*n* = 3) or not relevant population (*n* = 2). Ultimately, ten references met the inclusion criteria [8, 17–25]. For a detailed characterization of the included and excluded SRs, refer to Additional file 2. The ten SRs included were published between 2017 and 2025 and examined the diagnostic test accuracy of 23 readiness tests. All reviews were published in English, seven of them had registered protocols, and two [20, 21] provided enough information to conduct a meta-analysis using the available data.

The included studies examined critically ill adults aged 18 to 84, with mean APACHE scores ranging from 4 to 38 and mean SAPS II scores from 11 to 69. Participants required invasive mechanical ventilation for 24 h or more, with a mean duration of 2–58 days [17, 20, 24], and were recruited from various settings, including emergency departments and intensive care units (e.g., mixed, medical, cardiovascular, respiratory, burn/trauma, or neurological units). Reported admission reasons included the need for postoperative care, postoperative cardiovascular issues, exacerbated COPD, brain trauma or stroke, ARDS, or sepsis. Some reviews allowed the inclusion of studies involving tracheostomy users [8, 18, 20], morbidly obese patients [20], or individuals who experienced unplanned reintubation or extubation [18].

Exclusion criteria encompassed pregnant women, individuals with hemodynamic instability, tracheobronchial abnormalities, neuromuscular diseases, diaphragmatic paralysis, or long-term steroid use (more than 7 days). In addition, patients with a history of neck or laryngeal surgery, vocal cord paralysis, airway trauma or burns, and those with do-not-resuscitate orders were also excluded. The tests used to assess readiness for weaning from mechanical ventilation were conducted before, during, or after a spontaneous breathing trial with pressure support (5–8 cmH_2_O), T-piece, automatic tube compensation, continuous positive airway pressure (CPAP), or bilevel positive airway pressure (BiPAP). The clinical judgment was used to determine when participants were ready for weaning, while the reference standard for identifying failure involved the need for reintubation within the first 72 h, unsuccessful spontaneous breathing or the requirement for non-invasive ventilation within the first 48 h.

### Risk of bias of included SRs

Five reviews [8, 17, 18, 23, 25] were assessed as having an unclear risk of bias in the eligibility criteria domain. Although they had a published protocol, the information provided in the register did not confer sufficient elements regarding the appropriateness of eligibility criteria for the studies. Two systematic reviews [20, 22] were assessed as having a low risk of bias, because they followed a predefined protocol with clear objectives. The eligibility criteria were appropriate for the review question, and the limitations regarding information sources and study characteristics were established beforehand. Finally, three reviews [19, 21, 24] were assessed as high risk of bias, because they lacked a published research protocol.

Regarding study identification and selection domain, six reviews [17, 19, 20, 23–25] were assessed as having a high risk of bias due to their restriction to include published studies only. The search did not consider grey literature sources, was restricted by language, and in some circumstances, the search strategy did not provide sufficient information to ensure that as many relevant studies as possible were retrieved. The remaining [8, 18, 21, 22] reviews were assessed as having a low risk of bias for this domain, because the search included an appropriate range of databases, covered grey literature sources, the search strategy likely allowed for retrieval of as many studies as possible, and an effort was made to minimize bias when selecting studies.

**Fig. 2:**
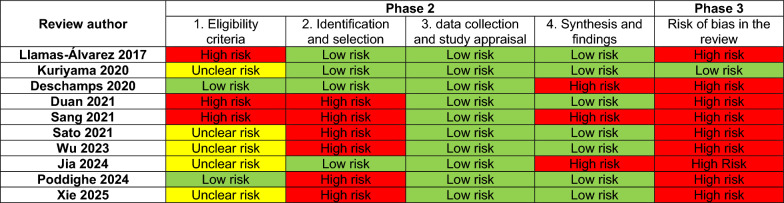
Risk of bias in included systematic reviews (ROBIS)

All SRs [8, 17–25] were assessed as having a low risk of bias for the data collection and study appraisal domain. The potential sources of error in data collection and analysis were minimized, all relevant studies retrieved were included in the synthesis, and the methodological evaluation was conducted using an appropriate and validated tool, minimizing biases during this process. Seven reviews [17, 18, 20, 21, 23–25] were appraised as having a low risk of bias for the synthesis and findings domain. Although these reviews did not assess the overall certainty of the evidence, they reported all predefined analyses, used appropriate synthesis methods for the data, and evaluated the risk of publication bias. In contrast, three reviews [8, 19, 22] were judged to have a high risk of bias, because their synthesis methods were not suitable for the data they analyzed or did not evaluate the risk of publication bias (Fig. 2).

#### Results of included SRs

The quality of the evidence was rated as very low to low, primarily due to limitations, such as risk of bias, indirectness, inconsistency, imprecision, and, in some cases, publication bias. Table 1 provides a summary of the diagnostic accuracy for each of the tests analyzed, along with an assessment of the evidence quality. For more detailed information, readers are encouraged to consult the supplementary material, which includes the certainty of the evidence and the corresponding “Summary of Findings” table (Additional file 3). In addition, the degree of publication overlap in this umbrella review was 8%, as determined by the CCA index, indicating a non-substantial overlap [14] (Additional file 4).

**Table 1:** Accuracy of tests evaluating readiness for liberation from mechanical ventilation in the adult population

Index test	Diagnostic estimates (95% CI)		Quality of the evidence (GRADE)
	Sensitivity	Specificity	
Cuff leak test [18]	0.66 (0.46–0.81)	0.88 (0.83–0.92)	
Cough peak flow [24]	0.76 (0.72–0.80)	0.75 (0.69–0.81)	
Semiquantitative cough strength score [24]	0.53 (0.41–0.64)	0.83 (0.74–0.89)	
Airway occlusion pressure at 100 ms [17]	0.86 (0.72–0.94)	0.58 (0.37–0.76)	
Rapid shallow breathing index [8]	0.60 (0.59–0.61)	0.68 (0.66–0.70)	
Maximal Inspiratory Pressure [20]	0.60 (0.44–0.74)	0.78 (0.68–0.85)	
Lung ultrasound [21]	0.94 (0.59–0.99)	0.87 (0.62–0.97)	
Diaphragmatic excursion [20]	0.74 (0.68–0.80)	0.80 (0.75–0.84)	
Diaphragmatic thickening fraction [20]	0.73 (0.65–0.80)	0.84 (0.77–0.89)	
Diaphragmatic rapid shallow breathing index [19]	0.84 (0.76–0.90)	0.87 (0.79–0.92)	
Diaphragmatic thickening fraction shallow breathing index [25]	0.85 (0.79–0.98)	0.65 (0.28–1.00)	
End-expiratory diaphragm thickness [20]	0.69 (0.50–0.83)	0.59 (0.32–0.82)	
End-inspiratory diaphragm thickness [20]	0.89 (0.59–0.98)	0.40 (0.05–0.90)	
Decrease in venous oxygen saturation [23]	0.83 (0.74–0.90)	0.88 (0.83–0.92)	
Change in Brain Natriuretic Peptide [22]	0.88 (0.83–0.92)	0.82 (0.73–0.89)	

GRADE Working Group grades of evidence

High quality: Further research is very unlikely to change our confidence in the estimate of effect

Moderate quality: Further research is likely to have an important impact on our confidence in the estimate of effect and may change the estimate

Low quality: Further research is very likely to have an important impact on our confidence in the estimate of effect and is likely to change the estimate

Very low quality: We are very uncertain about the estimate

#### Airway assessments tests

##### Cuff leak test

The cuff leak test evaluates laryngeal patency by deflating the endotracheal tube cuff to determine the presence of airflow around the tube, indicating whether the upper airway is unobstructed [18]. A meta-analysis [18] of 28 studies, which included 4493 patients, informed the diagnostic test accuracy of the cuff leak test. The median prevalence of weaning failure was 3%, and the reported cutoff for the test was the presence of an audible leak or, in a quantitative evaluation, an absolute volume > 110 mL or a proportion > 10% compared with the expiratory tidal volume. The mean sensitivity and specificity of all included studies were 0.66 (95% CI 0.46–0.81) and 0.88 (95% CI 0.83–0.92), for a mean LR + 5.59 (95% CI 3.48–8.98), LR −0.39 (95% CI 0.23–0.66), DOR 14.3 (95% CI 5.65–36.42), and AUC of 0.88 (95% CI 0.83–0.90). For a detailed characterization of the sources of heterogeneity, refer to Additional file 5 [26, 27]. The quality of the evidence was graded as very low ⨁◯◯◯ due to certain limitations related to risk of bias, inconsistency and imprecision [26, 27] (Additional file 3).

##### Cough peak flow (CPF)

CPF is a quantitative measure of expiratory muscle strength and airway clearance capacity. Reduced CPF has been associated with failed liberation from mechanical ventilation, whereas higher values predict successful weaning [24]. The results correspond to a SR [24] of nineteen studies, which included 2650 participants. The median prevalence of weaning failure was 18%, and the reported cutoff for cough peak flow was < 80 L/min. Pooled sensitivity and specificity for included studies were 0.76 (95% CI 0.72–0.80) and 0.75 (95% CI 0.69–0.81), with a mean LR + 2.89 (95% CI 2.36–3.54), LR- 0.37 (95% CI 0.30–0.45), DOR 8.91 (95% CI 5.96–13.3), and AUC of 0.79 (95% CI 0.75–0.82) (Additional file 5). The quality of the evidence was graded as low ⨁⨁◯◯ due to certain limitations related to risk of bias and inconsistency [26, 27] (Additional file 3).

##### Semiquantitative cough strength score (SCSS)

SCSS is a bedside tool used to assess cough efficacy on a scale from 0 (weak) to 5 (strong). It has been shown to predict reintubation risk after a planned extubation, with lower scores correlating with an increased likelihood of respiratory failure [24]. The results correspond to a SR [24] with twenty studies, which included 5543 participants. The median prevalence of weaning failure was 22%, and the reported cutoff for SCSS < 2 points. Pooled sensitivity and specificity for included studies were 0.53 (95% CI 0.41–0.64) and 0.83 (95% CI 0.74–0.89), with a mean LR + 2.50 (95% CI 1.93–3.25), LR −0.65 (95% CI 0.56–0.76), DOR 4.61 (95% CI 3.03–7.01), and AUC of 0.74 (95% CI 0.70–0.78) (Additional file 5). The quality of the evidence was graded as very low ⨁◯◯◯ due to certain limitations related to risk of bias, inconsistency and publication bias [26, 27] (Additional file 3).

#### Respiratory assessment test

##### Airway occlusion pressure at 100 ms (P0.1)

P0.1 is the negative inspiratory pressure exerted by the patient during the initial 0.1 s of an inspiratory effort against an occluded airway [17]. A meta-analysis [17] of twelve studies, which included 1088 participants, reported the diagnostic test accuracy of the P0.1 test. The median prevalence of weaning failure was 30%, and the reported cutoff for the test ranged between 2.3 and 5.0 cmH_2_O. The mean sensitivity and specificity of all included studies were 0.86 (95% CI 0.72–0.94) and 0.58 (95% CI 0.37–0.76), for a mean LR + 2.04 (95% CI 1.14–3.92), LR −0.24 (95% CI 0.08–0.75), DOR 20.0 (95% CI 1.63–247.15), and AUC of 0.81 (95% CI 0.77–0.84) (Additional file 5). The quality of the evidence was graded as very low ⨁◯◯◯ due to certain limitations related to risk of bias, inconsistency, and publication bias [26, 27] (Additional file 3).

##### Rapid shallow breathing index (RSBI)

RSBI is defined as the ratio of spontaneous respiratory rate (RR, breaths/min) to tidal volume (VT, liters), both measured during unassisted breathing [8]. A meta-analysis [8] of 79 studies, which included 13,170 participants, informed the diagnostic test accuracy of the RSBI test. The median prevalence of weaning failure was 29%, and the reported cutoff ranged between 44 and 130 breaths/min/L. The mean sensitivity and specificity of all included studies were 0.60 (95% CI 0.59–0.61) and 0.68 (95% CI 0.66–0.70), for a mean LR + 2.17 (95% CI 1.92–2.46), LR −0.31 (95% CI 0.26–0.36), DOR 9.23 (95% CI 7.06–12.08), and AUC of 0.81 (95% CI 0.79–0.83) (Additional file 5). The quality of the evidence was graded as very low ⨁◯◯◯ due to certain limitations related to risk of bias, indirectness, inconsistency, and publication bias [26, 27] (Additional file 3).

##### Maximal inspiratory pressure (MIP)

**Fig. 3:**
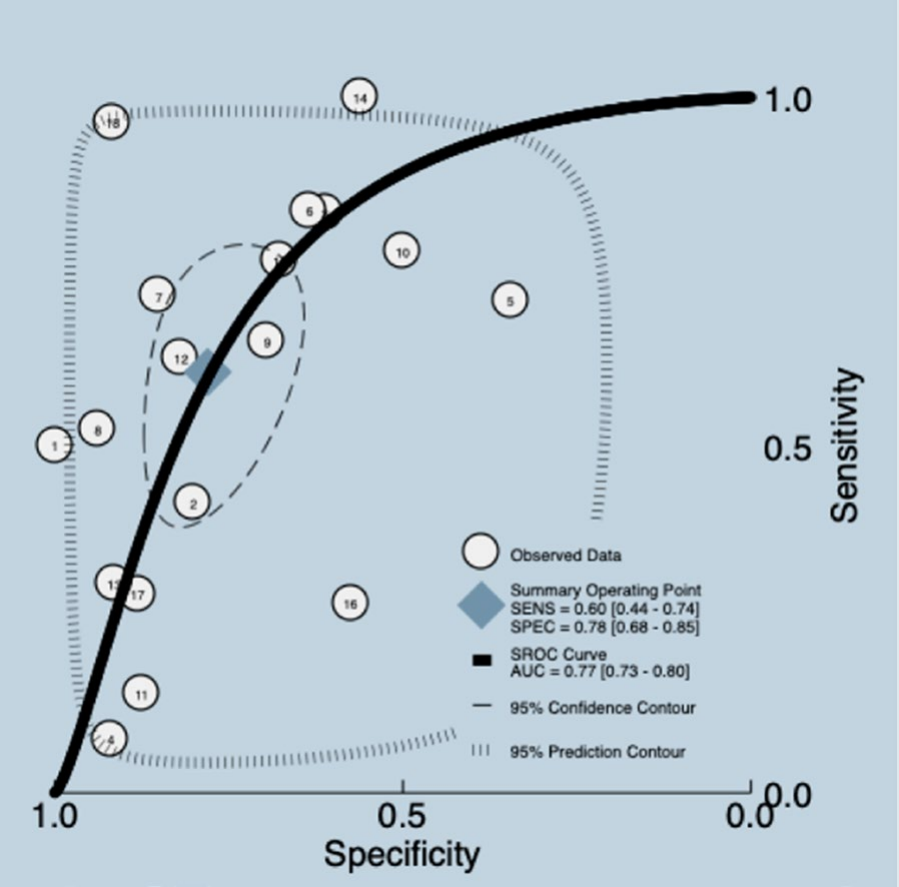
Summary ROC plot of tests: assessment of maximal inspiratory pressure diagnostic test accuracy

MIP quantifies the maximal negative pressure that the inspiratory muscles can generate against a closed airway, usually measured at residual lung volume [20]. A systematic review [20] included eighteen studies with 1107 participants, which reported the accuracy of the MIP. The median prevalence of weaning failure was 31%, and the reported cutoff for MIP 20–50 cmH_2_O. The data presented in the review were re-analyzed. Meta-analyses were implemented using the bivariate model, with a pooled sensitivity and specificity of 0.60 (95% CI 0.44–0.74) and 0.78 (95% CI 0.68–0.85), and a mean LR + 2.7 (95% CI 1.90–3.90), LR −0.51 (95% CI 0.35–0.74), DOR 5.0 (95% CI 3.0–10.0), and AUC of 0.77 (95% CI 0.73–0.80) (Fig. 3) (Additional file 5). The quality of the evidence was graded as very low ⨁◯◯◯ due to certain limitations related to risk of bias, indirectness and inconsistency [26, 27] (Additional file 3).

##### Lung ultrasound score (LUS)

**Fig. 4:**
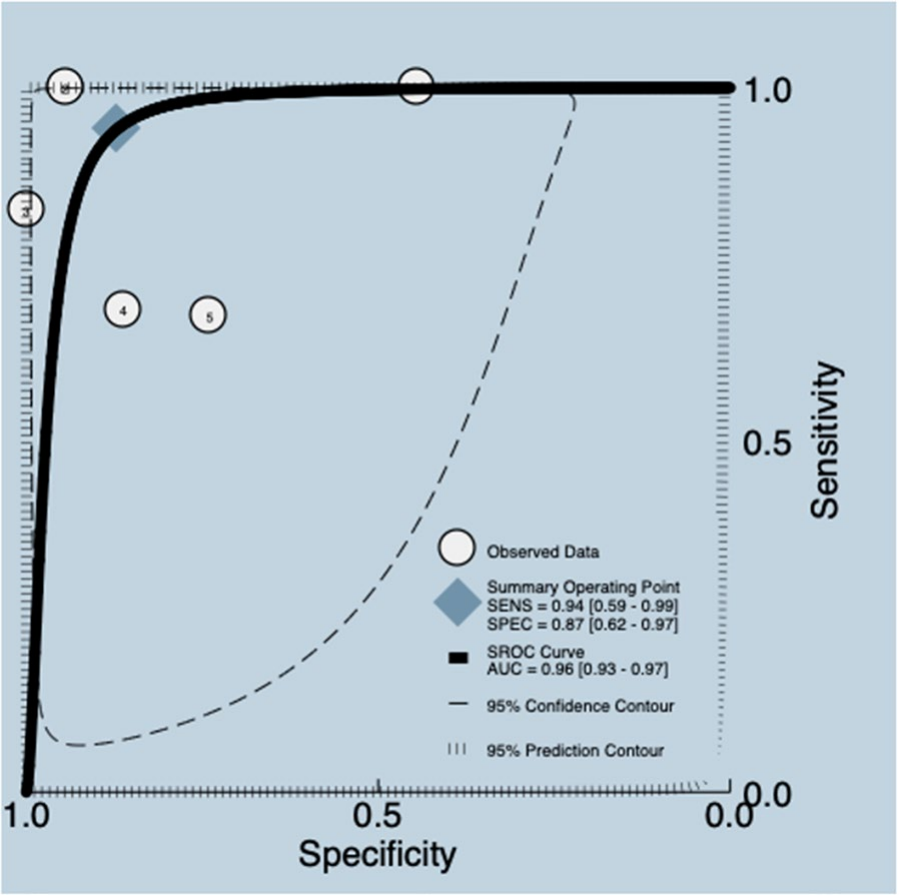
Summary ROC plot of tests: assessment of lung ultrasound score diagnostic test accuracy

LUS is a validated semiquantitative tool ranging from 0 to 36 points. It is derived by adding regional scores based on specific sonographic aeration patterns across 12 lung zones [21]. A systematic review [21] with five studies and 315 participants documented the diagnostic test accuracy of the LUS test. The median prevalence of weaning failure was 33%, and the reported cutoff was ≥ 17 points. The data presented in the review were re-analyzed. Meta-analyses were carried out, with a pooled sensitivity and specificity for included studies of 0.94 (95% CI 0.59–0.99) and 0.87 (95% CI 0.62–0.97), corresponding to a mean LR + 7.3 (95% CI 2.20–24.7), LR −0.07 (95% CI 0.01–0.65), DOR 108.0 (95% CI 8.0–1423.0), and AUC of 0.96 (95% CI 0.93–0.97) (Fig. 4) (Additional file 5). The quality of the evidence was graded as very low ⨁◯◯◯ due to certain limitations related to risk of bias, inconsistency and imprecision [26, 27] (Additional file 3).

#### Diaphragmatic assessments test

##### Diaphragmatic excursion (DE)

**Fig. 5:**
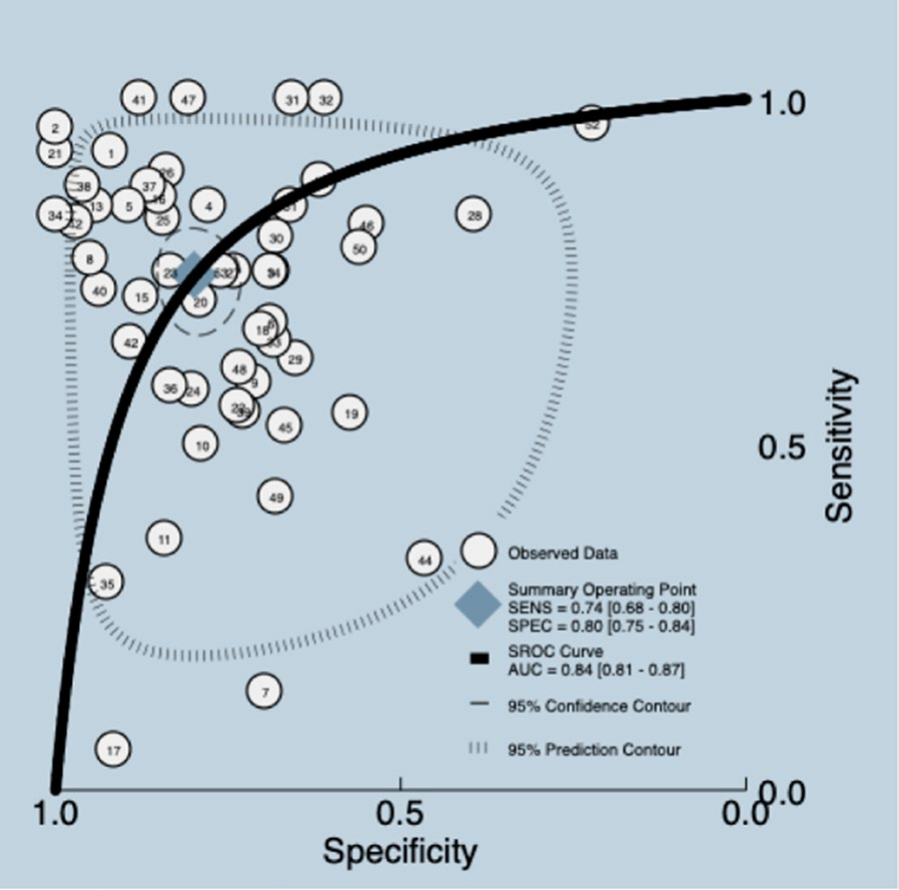
Summary ROC plot of tests: assessment of diaphragmatic excursion diagnostic test accuracy

DE is a sonographic parameter that quantifies the displacement of the diaphragm during breathing. It is an indirect marker of lung expansion and correlates positively with tidal volume [20].The results correspond to a SR [20] with 53 studies and a sample size of 3641 patients. The median prevalence of weaning failure was 32%, and the reported cutoff was 9–21 mm. The data presented in the review were re-analyzed. Meta-analyses were carried out, with a pooled sensitivity and specificity for included studies of 0.74 (95% CI 0.68–0.80) and 0.80 (95% CI 0.75–0.84) with a mean LR + 3.7 (95% CI 2.90–4.80), LR −0.32 (95% CI 0.25–0.41), DOR 11.0 (95% CI 7.0–18.0), and AUC of 0.84 (95% CI 0.81–0.87) (Fig. 5) (Additional file 5). The quality of the evidence was graded as very low ⨁◯◯◯ due to certain limitations related to risk of bias, indirectness, inconsistency and publication bias [26, 27] (Additional file 3).

##### Diaphragmatic thickening fraction (DTF)

**Fig. 6:**
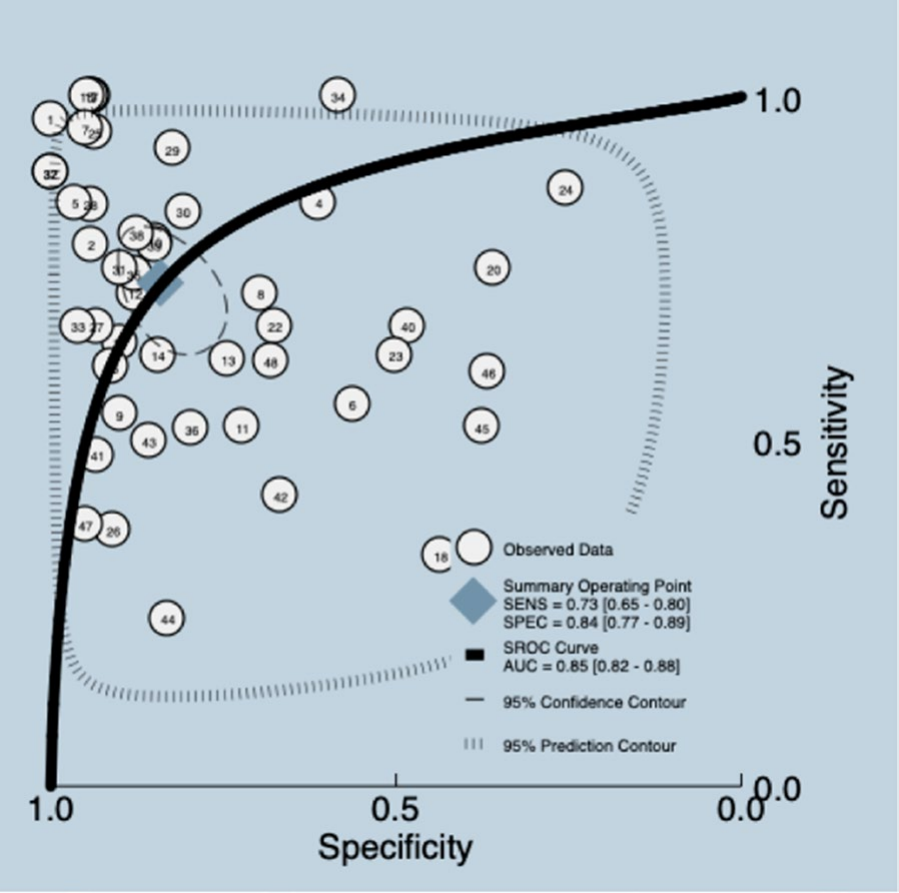
Summary ROC plot of tests: assessment of diaphragmatic thickening fraction diagnostic test accuracy

DTF is a sonographic index defined as (end-inspiratory thickness–end-expiratory thickness)/end-expiratory thickness. It reflects diaphragmatic contractility during spontaneous breathing [20]. One SR [20] retrieved 48 studies, including 3471 participants. The median prevalence of weaning failure was 31%, and the reported a cutoff ranged from 13% to 50%. The data presented in the review were re-analyzed. Meta-analyses were carried out. Aggregated sensitivity and specificity for included studies were 0.73 (95% CI 0.65–0.80) and 0.84 (95% CI 0.77–0.89), for a mean LR + 4.5 (95% CI 3.00–6.90), LR −0.32 (95% CI 0.24–0.44), DOR 14.0 (95% CI 7.0–28.0), and AUC of 0.85 (95% CI 0.82–0.88) (Fig. 6) (Additional file 5). The quality of the evidence was graded as very low ⨁◯◯◯ due to certain limitations related to risk of bias, inconsistency and publication bias [26, 27] (Additional file 3).

##### Diaphragmatic rapid shallow breathing index (D-RSBI)

D-RSBI is a modified version of the traditional RSBI. In this index, diaphragmatic excursion measured by ultrasound replaces spontaneous tidal volume in the RR/VT ratio. It is calculated as the respiratory rate (RR)-to-diaphragmatic displacement ratio during spontaneous breathing [19]. A meta-analysis [19] of nine studies, which included 568 patients, reported the diagnostic test accuracy of the D-RSBI test. The median prevalence of weaning failure was 30%, and the reported cutoff for the test ranged between 1.23 and 1.90 breaths/min/mm. The mean sensitivity and specificity of all included studies were 0.84 (95% CI 0.76–0.90) and 0.87 (95% CI 0.79–0.92), for a mean LR + 6.46 (95% CI 3.62–11.25), LR −0.18 (95% CI 0.11–0.30), DOR 35.8 (95% CI 32.91–37.50), and AUC of 0.88 (95% CI was not reported) (Additional file 5). The quality of the evidence was graded as low ⨁⨁◯◯ due to certain limitations related to inconsistency [26, 27] (Additional file 3).

##### Diaphragmatic thickening fraction rapid shallow breathing index (DTF–RSBI)

DTF–RSBI is a modified version of the traditional RSBI. In this index, diaphragmatic thickening fraction measured by ultrasound replaces spontaneous tidal volume in the RR/VT ratio [25]. A meta-analysis [25] of four studies, which included 414 patients, reported the diagnostic test accuracy of the DTF–RSBI test. The median prevalence of weaning failure was 33%, and the reported cutoff ranged between 48 and 85 breaths/min/percentage. The mean sensitivity and specificity of all included studies were 0.85 (95% CI 0.79–0.98) and 0.65 (95% CI 0.28–1.00), for a mean LR + 4.39 (95% CI 2.45–7.87), LR −0.19 (95% CI 0.05–0.63), DOR 23.1 (95% CI 12.49–49.00), and AUC of 0.88 (95% CI 0.85–0.90) (Additional file 5). The quality of the evidence was graded as low ⨁⨁◯◯ due to certain limitations related to inconsistency [26, 27] (Additional file 3).

##### End-expiratory diaphragm thickness (EeDT)

**Fig. 7:**
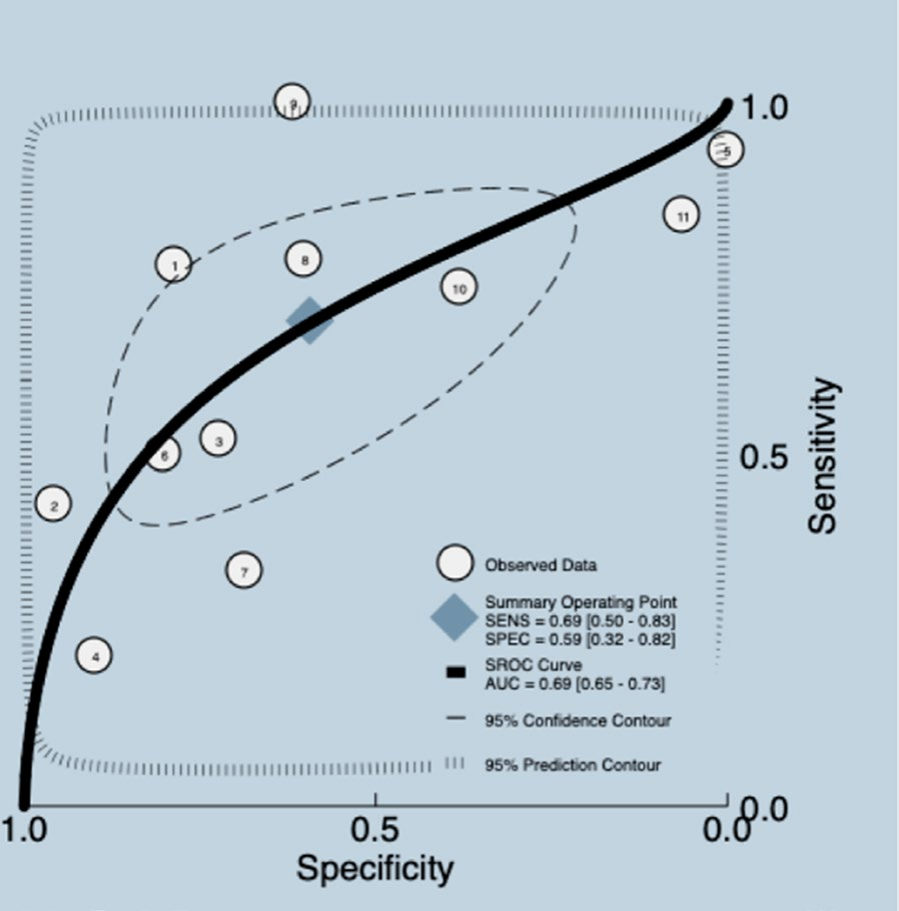
Summary ROC plot of tests: assessment of end-expiratory diaphragm thickness diagnostic test accuracy

EeDT is a sonographic parameter obtained at the end of a normal expiration using point-of-care ultrasound. It is commonly used to assess diaphragmatic function in critically ill patients [20]. A systematic review [20] with eleven studies and 617 participants assessed the accuracy of EeDT. The median prevalence of weaning failure was 34%, and the reported cutoff was 1.7–15.5 mm. The data presented in the review were re-analyzed. Meta-analyses were conducted. The pooled sensitivity and specificity for retrieved studies were 0.69 (95% CI 0.50–0.83) and 0.59 (95% CI 0.32–0.82), for a mean LR + 1.7 (95% CI 1.00–2.90), LR −0.53 (95% CI 0.34–0.81), DOR 3.0 (95% CI 1.0–7.0), and AUC of 0.69 (95% CI 0.65–0.73) (Fig. 7) (Additional file 5). The quality of the evidence was graded as low ⨁⨁◯◯ due to certain limitations related to risk of bias, inconsistency and imprecision [26, 27] (Additional file 3).

##### End-inspiratory diaphragm thickness (EiDT)

**Fig. 8:**
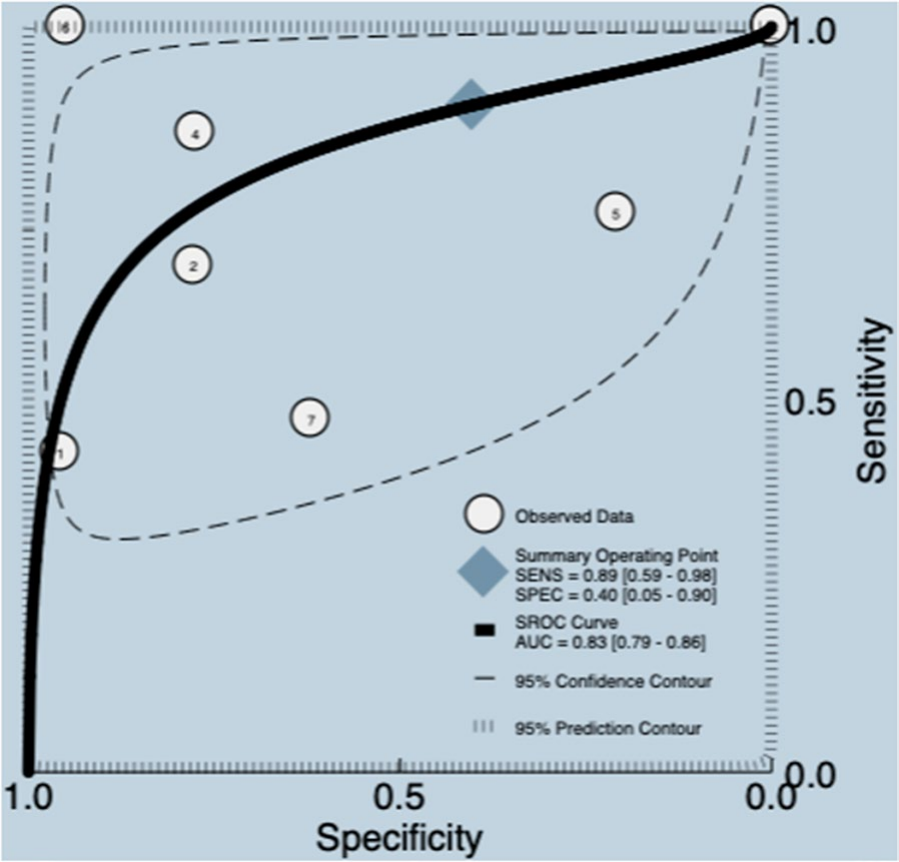
Summary ROC plot of tests: assessment of end-inspiratory diaphragm thickness diagnostic test accuracy

EiDT is a sonographic parameter obtained at the end of inspiration using point-of-care ultrasound, commonly used to assess diaphragmatic contractility in critically ill patients [20]. The results correspond to a SR [20] of eight studies, which included 464 participants. The median prevalence of weaning failure was 35%, and the reported cutoff was 3.4–21.0 mm. The data presented in the review were re-analyzed. Meta-analyses were carried out, with a pooled sensitivity and specificity for included studies of 0.89 (95% CI 0.59–0.98) and 0.40 (95% CI 0.05–0.90), with a mean LR + 1.5 (95% CI 0.6–3.80), LR −0.26 (95% CI 0.06–1.13), DOR 6.0 (95% CI 1.0–49.0), and AUC of 0.83 (95% CI 0.79–0.86) (Fig. 8) (Additional file 5). The quality of the evidence was graded as very low ⨁◯◯◯ due to certain limitations related to risk of bias, inconsistency and imprecision [26, 27] (Additional file 3).

#### Cardiovascular assessment tests

##### Decrease in venous oxygen saturation (ScvO2)

ScvO₂ is a hemodynamic parameter obtained from blood drawn through a central venous catheter. It reflects the global balance between oxygen delivery and oxygen consumption at tissue level and is commonly used to assess cardiovascular function in critically ill patients [23]. The results correspond to a SR [23] of five studies, which included 353 participants. The median prevalence of weaning failure was 28%, and the reported threshold for venous oxygen saturation was a reduction of more than 3.8%. The pooled sensitivity and specificity for included studies were 0.83 (95% CI 0.74–0.90) and 0.88 (95% CI 0.83–0.92), with a mean LR + 7.2 (95% CI 4.6–11.20), LR −0.19 (95% CI 0.12–0.31), DOR 38.0 (95% CI 17.0–86.0), and AUC of 0.92 (95% CI 0.90–0.94) (Additional file 5). The quality of the evidence was graded as low ⨁⨁◯◯ due to certain limitations related to risk of bias and inconsistency [26, 27] (Additional file 3).

##### Change in brain natriuretic peptide (ΔBNP)

Brain natriuretic peptide is a biomarker of myocardial stretch. A relative increase during a spontaneous breathing trial may indicate elevated left ventricular afterload and subclinical or overt pulmonary congestion. This variation has been proposed as a complementary predictor of successful weaning from mechanical ventilation [22]. The results correspond to a SR [22] of five studies, which included 257 participants. The median prevalence of weaning failure was 32%, and the reported cutoff was 13–20%. The pooled sensitivity and specificity for included studies were 0.88 (95% CI 0.83–0.92) and 0.82 (95% CI 0.73–0.89), with a mean LR + 5.1 (95% CI 3.1–8.4), LR −0.13 (95% CI 0.08–0.21), DOR 38.0 (95% CI 17.6–85.4), and AUC of 0.92 (95% CI 0.87–0.96) (Additional file 5). The quality of the evidence was graded as low ⨁⨁◯◯ due to certain limitations related to risk of bias [26, 27] (Additional file 3). For detailed information on the diagnostic accuracy of the other analyzed tests, please refer to Additional file 6 [20].

## Discussion

Based on the results of this overview of systematic reviews, tests used to assess readiness for weaning from mechanical ventilation in the adult population show weak to moderate diagnostic accuracy [28]. This performance translates into an average 6–47% chance of mistakenly indicating a successful weaning, and a 12–42% chance of mistakenly identifying failure during this process. Among the tests, the cuff leak test, LUS, D-RSBI, *ScvO2,* and BNP have a high to moderate diagnostic ability to rule in weaning failure [28], while other tests such as P0.1, RSBI, MIP, DE, DTF, DTF–RSBI, SCSS, and CPF suggest a weaker performance in this regard [1, 28, 29]. Conversely, the cuff leak test, P0.1, RSBI, MIP, DE, SCSS, CPF, and DTF exhibit weak diagnostic accuracy in ruling out unsuccessful weaning [1], in contrast to LUS, D-RSBI, DTF–RSBI, ScvO2, and BNP, which demonstrate high to moderate accuracy in this regard [28, 29]. The diagnostic accuracy of EeDT and EiDT is very poor, indicating for the time being, a limited or no discriminating ability to rule in or rule out weaning failure [1, 28, 29]. The findings of this review align with the views of some guideline development groups [2], which argue that the results of objective assessment tests are not definitive criteria but serve as a guide for decision-making [30]. The operational performance of each test is relatively modest but, used together, they help reduce uncertainty [1] regarding which patients are likely to be successfully withdrawn from mechanical ventilation [30]. Therefore, they should be considered as one of many factors involved in removing a patient from mechanical ventilation [1].

But why does the available evidence show limited performance? To answer this question, it must be remembered that although diagnostic tests provide the ability to discriminate between two health states [1], the result is far from being the true representation of a condition [1]. Recent publications highlight the importance of understanding and managing uncertainty to improve the diagnostic process [31–33]. Available literature alludes to uncertainty around a named diagnosis or disorder identified as causing the health problem [31]. However, uncertainty is pervasive throughout the entire diagnostic process [33], which unfolds over time. Therefore, understanding uncertainty across the evolving diagnostic process is essential for identifying ways to accept and handle uncertainty [32]. Consequently the clinical course of action will ultimately depend on the relative value given to the results (reflection of clinical experience) [27], the presence of additional sources of information [1] and, of course, the clinical scenario [34].

Thus, perhaps the reason why diagnostic accuracy may not be high is the fact that the population included in the studies represents only a part of the spectrum of the disease [34]. Although the included studies initially recruited patients with a wide range of disease severity (e.g., APACHE scores ranging from 4 to 38 and SAPS II scores from 11 to 69), it is important to note that the readiness tests analyzed are only applicable when the underlying pathology has been stabilized or adequately controlled. This is because only patients in relatively better condition are suitable candidates for the process of ventilator liberation. It is known that the diagnostic accuracy of the tests increases in the presence of severe disease (worse spectrum), considering that it is easier to detect “a large lesion than a small one” [27, 34].

Another factor that affects the operational performance, is the way in which the tests are applied and integrated [34]. Readiness tests are conducted after the results of the SBT are obtained, with the outcome determining whether the patient is eligible to continue in the mechanical liberation process. This creates an interdependence between readiness tests and the preceding one, which in this context is the SBT, serving as a conditional test. Although the final decision is based on the results observed at the end of the testing sequence, the conduct and interpretation of these readiness tests are inherently influenced by the outcomes of the prior SBT [34]. With the implementation of a parallel diagnostic strategy (as occurs with the readiness criteria) [34], one negative result in the presence of other positive results ultimately encourages the liberation from support. Using parallel tests allows an interpretation following the “believe the positive” OR rule, where the diagnosis is positive if any of the tests are, and only if the result is negative in all sources of information, is the test interpreted as negative [34].

However, this is not the only way in which performing a SBT can impact the accuracy of the readiness tests. SBT can be conducted using a T-piece after disconnecting the patient from the ventilator or with low-level pressure-support ventilation (PSV). Some studies indicate that the work of breathing required during a PSV trial is significantly lower than during a T-piece trial and less than that needed after liberation from mechanical ventilation. Consequently, while a PSV trial may facilitate a shorter time to liberation, it may also increase the risk of reintubation, because it tends to underestimate the actual work of breathing required post-extubation—particularly in patients with a high risk of failure, consequently impacting the operational performance of the tests. Finally, the lack of homogeneity in the cutoff points for each test can also explain, at least in part, the limited performance observed, since it impacts the intrinsic measurements of diagnostic accuracy (e.g., sensitivity, specificity, LR, DOR, and ROC) [27, 34]. Selecting a cutoff point for a diagnostic test is fundamentally a decision-making process, as measures of diagnostic accuracy—such as sensitivity versus specificity or positive versus negative likelihood ratios—are inversely related and vary depending on the chosen cutoff. Consequently, adjusting the cutoff involves a trade-off: a higher cutoff may reduce false positives but increase false negatives, while a lower cutoff can have the opposite effect. Evaluating the benefits and risks associated with potential inaccuracies is essential when determining the optimal cutoff, especially since absolute precision is rarely achievable. This consideration is crucial, as the selection of the cutoff directly influences the operational performance of the test and its clinical utility [27, 34].

This overview has some strengths [10]. It employed explicit and systematic methods to identify and analyze multiple SRs related to the research questions. The methods were pre-established and registered prior to execution. A comprehensive search was conducted in the most relevant databases, and encompassed gray literature, reference lists of included studies, and expert consultations, with no restrictions on language or date. Review selection, data extraction, and risk of bias assessments were performed in duplicate. To prevent double counting, a well-defined procedure was used to manage overlapping SRs [10]. The ROBIS [13] tool was implemented to assess the risk of bias in the included reviews. This overview synthesizes the results from the highest quality reviews, summarizing outcome data, and, in some cases, a meta-analysis was conducted based on the available data [10]. Finally, the GRADE approach was used to evaluate the strength of the evidence [27].

However, this overview also has limitations [10]. The included SRs have design and methodological flaws related to study eligibility criteria, identification, and selection [13]. In this sense, three SRs [19, 21, 24] were assessed as high risk of bias, because they lacked a published research protocol; six reviews [17, 19, 20, 23–25] were assessed as having a high risk of bias regarding the study identification and selection domain, due to their restriction to include published studies only; and three reviews [8, 19, 22] were judged to have a high risk of bias for the synthesis and findings domain, because their synthesis methods were not suitable for the data they analyzed or did not evaluate the risk of publication bias. These weaknesses may have resulted in some relevant studies being overlooked and decisions about included studies being influenced by their characteristics and findings [13]. In addition, SRs with a high risk of bias in the synthesis and findings domain, could potentially have resulted in an overestimation of the diagnostic test accuracy. This issue arises, because the methods used for the analysis do not account for the relationship between sensitivity and specificity and the heterogeneity in test accuracy, highlighting the need for the implementation of a hierarchical random-effects model [11].

Another limitation of the available evidence is the substantial heterogeneity observed for the tests analyzed. While heterogeneity is expected within diagnostic test studies due to the use of different cutoff points, differences in SBT and definition of weaning failure, variability in the clinical setting, and consideration of the disease spectrum, heterogeneity for the analyzed variables could not be explained. This aspect seriously limits our confidence in the certainty of the effect (operational performance) [32, 33]. As for the precision of the results, there is uncertainty regarding the true magnitude of diagnostic test accuracy for some tests. This limitation is evident due to the width of the confidence intervals around point estimates for sensitivity, specificity, or both.

In addition, the degree of publication overlap in this umbrella review was 8%, as calculated using the CCA index (see Supplementary Material Table S4). This overlap indicates that the evidence base may not be entirely independent, with some primary studies potentially included multiple times. Such redundancy could lead to an overestimation of the overall strength of the findings. Therefore, caution is advised when applying these results in clinical practice. These considerations ultimately lead to the quality of the evidence being graded as low or very low, not only due to limitations in risk of bias, but also to indirectness, inconsistency, imprecision and, in some cases, publication bias [16].

Despite its limitations, this overview of systematic reviews has implications for both practice and research. Given the potential negative outcomes of improper ventilator liberation and the limitations of the current evidence, the readiness tests should be incorporated as part of a comprehensive diagnostic strategy. This strategy should involve accurately identifying patients with the highest pretest probability of successful extubation—considering factors such as evidence of improvement in the underlying cause of respiratory failure, adequate oxygenation, hemodynamic stability, and the ability to initiate inspiratory effort [35]—to then proceed to implement and interpret the readiness tests in parallel. This approach will allow overcoming the limitations offered by individual operational performance [32]. Physicians should integrate the results of physiological, ultrasound, and paraclinical measures to minimize uncertainty in deciding which patients should progress toward liberation from mechanical ventilation, always considering individual patient needs, to ensure personalized healthcare.

In this context, it is critical to distinguish between readiness for ventilator liberation and extubation, two conceptually and clinically distinct stages in discontinuing mechanical ventilation. According to the most recent guideline from the American Association for Respiratory Care [36], ventilator liberation is defined by the patient's ability to sustain spontaneous breathing, typically assessed through a spontaneous breathing trial (SBT). In contrast, extubation readiness involves a broader evaluation of airway patency, secretion management, cough strength, and upper airway protection. Evidence shows that a significant proportion of patients who pass an SBT may still experience extubation failure due to these non-ventilatory factors [37–39]. Therefore, the diagnostic tests incorporated in our review—encompassing physiological respiratory measures (e.g., RSBI, MIP, and P0.1), bedside functional assessments (such as cough peak flow and semiquantitative cough strength scores), and ultrasound-based evaluations (e.g., diaphragmatic excursion, thickening fraction, and lung ultrasound)—should be implemented and interpreted in parallel with the aim of supporting extubation readiness assessment.

While these tests are increasingly adopted in clinical practice and supported by growing evidence, they are not yet formally incorporated into the main international clinical practice guidelines. For instance, the ATS/ACCP guideline [40] and the AARC guideline [36] focus on the role of spontaneous breathing trials and the cuff leak test, without recommending the routine use of RSBI, P0.1, MIP, or ultrasound-derived indices. The joint ERS/ATS/SCCM consensus statement [2] acknowledges the potential utility of indices such as RSBI and MIP but stops short of providing strong recommendations. Moreover, the French guidelines issued by SFAR and SRLF [41] concentrate primarily on airway protection and extubation failure risk, without addressing other clinically relevant tests, such as cough strength or respiratory and diaphragmatic ultrasound. This discrepancy reflects a gap between the growing evidence supporting these diagnostic tests and their integration into clinical protocols. Our findings contribute to bridging this gap by synthesizing the diagnostic accuracy of emerging tests that could improve patient selection and personalized decision-making for extubation. These results may serve as a foundation for future clinical guideline updates and the broader implementation of multimodal assessment strategies in the ICU.

It is essential to promote more comprehensive and high-quality research evaluating the operational performance of tests used to assess readiness for weaning from mechanical ventilation in adults. These studies should particularly focus on subgroups, where test accuracy may vary significantly, such as elderly patients, individuals with cardiac comorbidities, and those requiring prolonged ventilation. In addition, it is crucial for future research to standardize cutoff values and protocols, as well as to conduct direct comparisons between different assessment methods—including airway, respiratory, and diaphragmatic tests—to determine their relative diagnostic accuracy. Future SRs should include well-defined eligibility criteria for primary studies, expanding the search to gray literature without restrictions on language or publication date. Moreover, employing robust methods for information synthesis and assessing publication bias is crucial to address unresolved uncertainties and, in particular, to confirm the potential role of lung ultrasound (LUS) and the accuracy of various functional parameters, such as parasternal intercostal thickening fraction, phrenic nerve stimulation, and diaphragmatic electrical activity. Furthermore, it is crucial to evaluate how proportional ventilation modes and artificial intelligence will affect both the assessment and progression of the liberation from mechanical ventilation process, and what will be their impact on the diagnostic accuracy of readiness tests.

## Conclusions

The accuracy of tests used to assess readiness for weaning from mechanical ventilation in adults varies considerably. On average, these tests yield a 6–47% chance of incorrectly indicating successful weaning, and a 12–42% chance of mistakenly suggesting failure. Given the potential negative consequences of improper liberation from mechanical ventilation, physicians should integrate the results of physiological, ultrasound, and paraclinical measures to minimize uncertainty in deciding which patients should progress toward ventilator weaning, always considering individual patient needs to ensure personalized healthcare.

## Supplementary Information

Additional file 1.

Additional file 2.

Additional file 3.

Additional file 4.

Additional file 5.

Additional file 6.

## Data Availability

The data set analyzed in the current study is available as supplementary material.
