# Supplementary material for: Diagnostic accuracy of tests for assessing readiness for liberation from mechanical ventilation in adults: an overview of reviews
Source: J Intensive Care. 2026 Jan 14;14:14. doi: 10.1186/s40560-026-00848-9 (PMC12874667; doi:10.1186/s40560-026-00848-9)
Supplement: Supplementary file 1 — Additional file 1. [file 40560_2026_848_MOESM1_ESM.docx]

| **Electronic Search Report #1** | |
| --- | --- |
| **Database** | MEDLINE(ALL) |
| **Platform** | Ovid |
| **Search date** | 10-Nov-2025 |
| **Language restriction** | None |
| **Other limits** | None |
| **Search strategy** | 1. exp Ventilator Weaning/ (4906) 2. (weaning adj5 (ventilat$ or respirator)).tw. (4487) 3. ("wean off" adj5 (vent$ or respirat$)).tw. (74) 4. exp Airway Extubation/ (2949) 5. extubation$.tw. (16053) 6. detubation.tw. (10) 7. exp Respiration, Artificial/ (95015) 8. (artificial adj5 (respirat$ or ventilat$)).tw. (6006) 9. (mechanical adj5 (respirat$ or ventilat$)).tw. (71331) 10. (controlled adj5 (respirat$ or ventilat$)).tw. (7066) 11. "intubation".tw. (62483) 12. or/7-11 (194586) 13. withdrawal.tw. (113647) 14. discontinuation.tw. (74112) 15. disconnect.tw. (4007) 16. or/13-15 (186870) 17. 12 and 16 (2962) 18. or/1-6,17 (24402) 19. exp Tidal Volume/ (11283) 20. (tidal adj3 volume$).tw. (17722) 21. (tidal adj1 air).tw. (74) 22. exp Pulmonary Ventilation/ (48201) 23. (pulmonary adj5 ventilation).tw. (7975) 24. (lung adj5 ventilation).tw. (13072) 25. (alveol$ adj5 ventilation).tw. (2485) 26. ((respiratory or expiratory) adj5 airflow).tw. (1755) 27. (ventilation adj5 (test$ or asynchronous or perfusion or rate)).tw. (14247) 28. (pulmonary adj5 aeration).tw. (81) 29. (negative adj5 inspirat$ adj5 force).tw. (59) 30. (negative adj5 press$).tw. (18844) 31. nif.tw. (2435) 32. (occlusion adj5 pressure).tw. (3658) 33. "p 0.1".tw. (92477) 34. (tobin adj5 index).tw. (10) 35. (rapid adj5 shallow adj5 breath$).tw. (688) 36. rsbi.tw. (215) 37. exp Diaphragm/dg [Diagnostic Imaging] (2370) 38. (diaphragm$ adj5 (ultraso$ or sonographic or imaging)).tw. (1676) 39. (cuff adj5 leak adj5 test).tw. (92) 40. (weaning adj5 index).tw. (190) 41. exp Vital Capacity/ (29789) 42. (vital adj5 capacit$).tw. (23092) 43. or/19-42 (230638) 44. 18 and 43 (3281) 45. exp "Sensitivity and Specificity"/ (698747) 46. sensitivity.tw. (1138683) 47. specificity.tw. (631444) 48. ((pre-test or pretest) adj probability).tw. (3216) 49. post-test probability.tw. (820) 50. predictive value$.tw. (160453) 51. likelihood ratio$.tw. (22463) 52. or/45-51 (1959263) 53. Incidence/ (327619) 54. exp Mortality/ (446943) 55. Follow-Up Studies/ (721337) 56. prognos$.tw. (943711) 57. predict$.tw. (2441549) 58. cours$.tw. (772821) 59. or/53-58 (4829272) 60. 52 or 59 (6236540) 61. 44 and 60 (1240) |
| **Records identified through database searching** | 1240 |
| **After duplicate records removed** | 636 |

| **Electronic Search Report #2** | |
| --- | --- |
| **Database** | EMBASE |
| **Platform** | Embase.com |
| **Search date** | 10-Nov-2025 |
| **Language restriction** | None |
| **Other limits** | None |
| **Search strategy** | 1. 'ventilator weaning'/exp (5563) 2. (weaning NEAR/5 (ventilat* OR respirator)):ti,ab (7974) 3. ('wean off' NEAR/5 (vent* OR respirat*)):ti,ab (192) 4. 'extubation'/exp (35106) 5. extubation*:ti,ab (28740) 6. detubation:ti,ab (26) 7. 'artificial ventilation'/exp (349094) 8. (artificial NEAR/5 (respirat* OR ventilat*)):ti,ab (8064) 9. (mechanical NEAR/5 (respirat* OR ventilat*)):ti,ab (122207) 10. (controlled NEAR/5 (respirat* OR ventilat*)):ti,ab (10765) 11. 'intubation':ti,ab (106728) 12. #7 OR #8 OR #9 OR #10 OR #11 (446570) 13. withdrawal:ti,ab (175169) 14. discontinuation:ti,ab (144883) 15. disconnect:ti,ab (5548) 16. #13 OR #14 OR #15 (315453) 17. #12 AND #16 (8347) 18. #1 OR #2 OR #3 OR #4 OR #5 OR #6 OR #17 (57524) 19. 'tidal volume'/exp (31739) 20. (tidal NEAR/3 volume*):ti,ab (27318) 21. (tidal NEAR/1 air):ti,ab (93) 22. 'lung ventilation'/exp (42118) 23. (pulmonary NEAR/5 ventilation):ti,ab (11470) 24. (lung NEAR/5 ventilation):ti,ab (20542) 25. (alveol* NEAR/5 ventilation):ti,ab (3621) 26. ((respiratory OR expiratory) NEAR/5 airflow):ti,ab (2726) 27. (ventilation NEAR/5 (test* OR asynchronous OR perfusion OR rate)):ti,ab (21526) 28. (pulmonary NEAR/5 aeration):ti,ab (161) 29. (negative NEAR/5 inspirat* NEAR/5 force):ti,ab (232) 30. (negative NEAR/5 press*):ti,ab (25008) 31. nif:ti,ab (2743) 32. (occlusion NEAR/5 pressure):ti,ab (4860) 33. 'p 0.1':ti,ab (34625) 34. (tobin NEAR/5 index):ti,ab (33) 35. (rapid NEAR/5 shallow NEAR/5 breath*):ti,ab (1111) 36. rsbi:ti,ab (480) 37. 'diaphragm'/exp AND 'ultrasound'/exp (1496) 38. (diaphragm* NEAR/5 (ultraso* OR sonographic OR imaging)):ti,ab (2967) 39. (cuff NEAR/5 leak NEAR/5 test):ti,ab (179) 40. (weaning NEAR/5 index):ti,ab (336) 41. 'vital capacity'/exp (12102) 42. (vital NEAR/5 capacit*):ti,ab (34768) 43. #19 OR #20 OR #21 OR #22 OR #23 OR #24 OR #25 OR #26 OR #27 OR #28 OR #29 OR #30 OR #31 OR #32 OR #33 OR #34 OR #35 OR #36 OR #37 OR #38 OR #39 OR #40 OR #41 OR #42 (212046) 44. #18 AND #43 (7264) 45. 'sensitivity and specificity'/exp (571787) 46. sensitivity:ti,ab (1509754) 47. specificity:ti,ab (843650) 48. (('pre test' OR pretest) NEAR/5 probability):ti,ab (6282) 49. 'post-test probability':ti,ab (1176) 50. (predictive NEAR/1 value*):ti,ab (244131) 51. (likelihood NEAR/1 ratio*):ti,ab (30835) 52. 'diagnostic accuracy'/exp (357504) 53. #45 OR #46 OR #47 OR #48 OR #49 OR #50 OR #51 OR #52 (2395149) 54. 'morbidity'/de (470910) 55. 'mortality'/exp (1644573) 56. 'follow up'/de (2569067) 57. prognos*:ti,ab (1440317) 58. predict*:ti,ab (3303341) 59. cours*:ti,ab (1140650) 60. #54 OR #55 OR #56 OR #57 OR #58 OR #59 (8353573) 61. #53 OR #60 (9974972) 62. #44 AND #61 (3220) 63. #44 AND #61 AND [embase]/lim (2680) |
| **Records identified through database searching** | 2680 |
| **After duplicate records removed** | 2445 |

| **Electronic Search Report #3** | |
| --- | --- |
| **Database** | Cochrane Central Register of Controlled Trials |
| **Platform** | Ovid-PaulinaG |
| **Search date** | 10-Nov-2025 |
| **Language restriction** | None |
| **Other limits** | None |
| **Search strategy** | 1. exp Ventilator Weaning/ (650) 2. (weaning adj5 (ventilat$ or respirator)).tw. (1449) 3. ("wean off" adj5 (vent$ or respirat$)).tw. (22) 4. exp Airway Extubation/ (464) 5. extubation$.tw. (11450) 6. detubation.tw. (20) 7. exp Respiration, Artificial/ (9245) 8. (artificial adj5 (respirat$ or ventilat$)).tw. (456) 9. (mechanical adj5 (respirat$ or ventilat$)).tw. (15758) 10. (controlled adj5 (respirat$ or ventilat$)).tw. (3064) 11. "intubation".tw. (22944) 12. or/7-11 (43273) 13. withdrawal.tw. (24269) 14. discontinuation.tw. (26510) 15. disconnect.tw. (195) 16. or/13-15 (48686) 17. 12 and 16 (1289) 18. or/1-6,17 (13657) 19. exp Tidal Volume/ (1246) 20. (tidal adj3 volume$).tw. (4704) 21. (tidal adj1 air).tw. (16) 22. exp Pulmonary Ventilation/ (8390) 23. (pulmonary adj5 ventilation).tw. (1341) 24. (lung adj5 ventilation).tw. (2966) 25. (alveol$ adj5 ventilation).tw. (326) 26. ((respiratory or expiratory) adj5 airflow).tw. (218) 27. (ventilation adj5 (test$ or asynchronous or perfusion or rate)).tw. (2524) 28. (pulmonary adj5 aeration).tw. (22) 29. (negative adj5 inspirat$ adj5 force).tw. (16) 30. (negative adj5 press$).tw. (2670) 31. nif.tw. (109) 32. (occlusion adj5 pressure).tw. (770) 33. "p 0.1".tw. (14273) 34. (tobin adj5 index).tw. (11) 35. (rapid adj5 shallow adj5 breath$).tw. (165) 36. rsbi.tw. (85) 37. exp Diaphragm/dg [Diagnostic Imaging] (3) 38. (diaphragm$ adj5 (ultraso$ or sonographic or imaging)).tw. (378) 39. (cuff adj5 leak adj5 test).tw. (24) 40. (weaning adj5 index).tw. (38) 41. exp Vital Capacity/ (3828) 42. (vital adj5 capacit$).tw. (6450) 43. or/19-42 (41256) 44. 18 and 43 (1570) |
| **Records identified through database searching** | 1570 |
| **After duplicate records removed** | 1322 |

| **Electronic Search Report #4** | |
| --- | --- |
| **Database** | CINAHL |
| **Platform** | EBSCO |
| **Search date** | 10-Nov-2025 |
| **Language restriction** | None |
| **Other limits** | None |
| **Search strategy** | 1. MH "Ventilator Weaning"  (3148) 2. TI ( (weaning N5 (ventilat* OR respirator)) ) OR AB ( (weaning N5 (ventilat* OR respirator)) ) (2005) 3. TI ( ("wean off" N5 (vent* OR respirat*)) ) OR AB ( ("wean off" N5 (vent* OR respirat*)) ) (21) 4. MH "Airway Extubation"  (1632) 5. TI extubation* OR AB extubation* (5130) 6. TI detubation OR AB detubation  (0) 7. MH "Respiration, Artificial" OR TI ( (artificial N4 (respirat* OR ventilat*)) ) OR AB ( (artificial N4 (respirat* OR ventilat*)) ) OR TI ( (mechanical N4 (respirat* OR ventilat*)) ) OR AB ( (mechanical N4 (respirat* OR ventilat*)) ) OR TI ( (controlled N4 (respirat* OR ventilat*)) ) OR AB ( (controlled N4 (respirat* OR ventilat*)) ) OR TI intubation OR AB intubation (56303) 8. TI withdrawal OR AB withdrawal OR TI discontinuation OR AB discontinuation OR TI disconnect OR AB disconnect  (42885) 9. S7 AND S8 (890) 10. S1 OR S2 OR S3 OR S4 OR S5 OR S6 OR S9 (9338) 11. MH "Tidal Volume"  (3448) 12. TI (tidal N2 volume*) OR AB (tidal N2 volume*) (4259) 13. TI (tidal N0 air) OR AB (tidal N0 air) (3) 14. MH "Pulmonary Ventilation"  (10318) 15. TI (pulmonary N4 ventilation) OR AB (pulmonary N4 ventilation) (1400) 16. TI (lung N4 ventilation) OR AB (lung N4 ventilation) (3359) 17. TI (alveol* N4 ventilation) OR AB (alveol* N4 ventilation) (390) 18. TI ( ((respiratory OR expiratory) N4 airflow) ) OR AB ( ((respiratory OR expiratory) N4 airflow) ) (328) 19. TI ( (ventilation N4 (test* OR asynchronous OR perfusion OR rate)) ) OR AB ( (ventilation N4 (test* OR asynchronous OR perfusion OR rate)) ) (3308) 20. TI (pulmonary N4 aeration) OR AB (pulmonary N4 aeration) (26) 21. TI (negative N4 inspirat* N4 force) OR AB (negative N4 inspirat* N4 force) (28) 22. TI (negative N4 press*) OR AB(negative N4 press*) (4961) 23. TI nif OR AB nif (57) 24. TI (occlusion N4 pressure) OR AB (occlusion N4 pressure) (802) 25. TI "p 0.1" OR AB "p 0.1" (2571) 26. TI (tobin N4 index) OR AB (tobin N4 index) (8) 27. TI (rapid N4 shallow N4 breath*) OR AB (rapid N4 shallow N4 breath*) (201) 28. TI rsbi OR AB rsbi  (79) 29. MH "Diaphragm" AND MH "Ultrasonography" (221) 30. TI ( diaphragm* N4 (ultraso* OR sonographic OR imaging) ) OR AB ( diaphragm* N4 (ultraso* OR sonographic OR imaging) ) (552) 31. TI (cuff N4 leak N4 test) OR AB (cuff N4 leak N4 test) (52) 32. TI (weaning N4 index) OR AB (weaning N4 index) (84) 33. MH "Vital Capacity"  (5123) 34. TI (vital N4 capacit*) OR AB (vital N4 capacit*) (4657) 35. S34 OR S33 OR S32 OR S31 OR S30 OR S29 OR S28 OR S27 OR S26 OR S25 OR S24 OR S23 OR S22 OR S21 OR S20 OR S19 OR S18 OR S17 OR S16 OR S15 OR S14 OR S13 OR S12 OR S11 (28182) 36. S35 AND S10 (1190) 37. MH "Sensitivity and Specificity"  (102772) 38. TI sensitivity OR AB sensitivity  (155328) 39. TI specificity OR AB specificity  (79265) 40. TI ( ((pre-test OR pretest) N4 probability) ) OR AB ( ((pre-test OR pretest) N4 probability) ) (1065) 41. TI post-test probability OR AB post-test probability  (323) 42. TI predictive value* OR AB predictive value* (37467) 43. TI likelihood ratio* OR AB likelihood ratio*  (7199) 44. S43 OR S42 OR S41 OR S40 OR S39 OR S38 OR S37 (248591) 45. MH Incidence (83430) 46. MH Mortality  (36775) 47. MH "Follow-Up Studies"  (6984) 48. TI prognos* OR AB prognos* (162161) 49. TI predict* OR AB predict* (513480) 50. TI cours* OR AB cours* (150205) 51. S50 OR S49 OR S48 OR S47 OR S46 OR S45 (852569) 52. S51 OR S44 (1014391) 53. S52 AND S36 (420) |
| **Records identified through database searching** | 420 |
| **After duplicate records removed** | 197 |
