# Supplementary material for: Diagnostic accuracy of tests for assessing readiness for liberation from mechanical ventilation in adults: an overview of reviews
Source: J Intensive Care. 2026 Jan 14;14:14. doi: 10.1186/s40560-026-00848-9 (PMC12874667; doi:10.1186/s40560-026-00848-9)
Supplement: Supplementary file 2 — Additional file 2. [file 40560_2026_848_MOESM2_ESM.docx]

**Additional File 2 Characterization of the included and excluded SRs**

**Table B1. Characteristics of Included Reviews: Design and Development**

| **REVIEW** | **OBJECTIVE** | **INCLUSION CRITERIA** | | | | |
| --- | --- | --- | --- | --- | --- | --- |
|  |  | **POPULATION** | **INTERVENTION** | **COMPARISONS** | **STUDY DESIGN** | **CONSULTED DATABASES** |
| Sato  2021 | Assess the predictive value of P0.1 in predicting successful weaning and liberation from mechanical ventilation | Patients over 18 years of age on mechanical ventilation for at least 24 hours  Age: 52 – 70 years  Females: 45-100%  APACHE II: 16 – 29  SAPS II: 11 – 39  Duration of mechanical ventilation: 6,5 – 58 days  Failure of extubating/weaning: 16,31 – 53,83 % | **P0.1**  P0.1 with cut-off point > 2,3 – 5,5 cmH2O, performed before, during or after spontaneous ventilation test with PS 5 - 7 cmH2O, T-piece or Automatic tube compensation. | Reintubation, use of non-invasive ventilation or death within 24 – 48 hours after extubation or failure of SBT | Diagnostic accuracy studies | MEDLINE, Cochrane Central Register of Controlled Trials, EMBASE |
| Kuriyama  2020 | Assess the diagnostic accuracy of the cuff leak test for predicting post-extubation airway obstruction. | Patients over 18 years of age on mechanical for at least 12 hours  Age: 56 – 67 years  Female: 16.4 – 47,4%  APACHE II: 17 – 19,7  SAPS II: 26 – 46  Use of corticosteroids 9/12 studies (23 – 66%)  Duration of mechanical ventilation: 2 – 12,9 days  Failure of extubation/weaning: 0,57 – 18 % | **CUFF LEAK TEST**  1.Quantitative tests: tidal volume variation from < 1325 – 50 ml or 15,5 -57%  2.Qualitative tests: presence or absence of leak around the tube.  Performed during assist-control mechanical ventilation (Vt 6 – 12 ml/Kg, RR 20 bpm) or spontaneous mode (T-piece, PSV or CPAP) | Reintubation or tracheostomy within at least 24 hours of extubation or laryngeal edema ending in reintubation | Diagnostic accuracy studies | MEDLINE, EMBASE, Scopus, ISI Web of Science, the Cochrane Library |
| Jia  2024 | Assess the predictive value of the rapid shallow breathing index for extubation outcomes. | Patients over 18 years of age, on mechanical ventilation for at least 24 – 72 hours or ventilated through a tracheostomy tube  Age: 35 – 79 years  Female: 7 – 84%  APACHE II 8 – 38  SAPS II: 36 – 66  Duration of mechanical ventilation: 1 – 28 days  Failure of extubation/Weaning: 6,12 – 67% | **RSBI**  RSBI with cut-off point > 34,5 - 43.5 - 130 respirations/min/L, performed during or after a spontaneous ventilation test with T-Piece, CPAP or PSV (PS 5 -10 cmH2O, PEEP 0 – 5 cmH2O) | Reintubation, use of non-invasive ventilation, need for tracheostomy or death within 24 – 72 hours after extubation or failure of SBT | Diagnostic accuracy studies | EMBASE, CENTRAL y PUBMED, CNKI, WangFang, VIP, SinoMed |
| Sang  2021 | Assess the predictive value of diaphragmatic rapid shallow breathing index (D-RSBI) of weaning outcome | Patients over 18 years of age, on mechanical ventilation for at least 24 – 48 hours  Age 36 – 76 years  Female: 27 – 71 %  APACHE II: 8 – 22  SAPS II: 37 – 40  Duration of mechanical ventilation: 5 days  Failure of extubation/Weaning: 19 – 40% | **(US-RSBI)**  D-RSBI with cut-off point > 1.13 – 1.9, performed before or during spontaneous ventilation test with T-Piece, PSV (PS 5 – 8 cmH2O, PEEP ≤ 5 cmH2O) | Reintubation, use of non-invasive ventilation or death within 48 hours after extubation or failure of SBT | Diagnostic accuracy studies | Pubmed, Cochrane Library, Embase, CNKI and Wanfang Data |
| Poddighe  2024 | Estimate and compare the accuracy of bedside respiratory muscle assessments to predict weaning outcomes, | 1. **Maximal inspiratory pressure (MIP)**   Patients over 18 years of age, on mechanical ventilation for at least 24 hours up 7 days  Age: 43 – 79 years  Female: 26 – 66 %  APACHE II: 4 – 25  SAPS II: 11 – 76  Duration of mechanical ventilation: 3 – 30 days  Failure of extubation/Weaning: 7 – 44%   1. **Diaphragm excursion (DE)**   Patients over 18 years of age, on mechanical ventilation for at least 24 hours up 30 days  Age: 37 – 84 years  Female: 25 – 58 %  APACHE II: 12 – 39  SAPS II: 34 – 76  Duration of mechanical ventilation: 2 – 30 days  Failure of extubation/Weaning: 6 – 80%   1. **Diaphragm thickening fraction (DTF)**   Patients over 16 years of age, on mechanical ventilation for at least 24 hours up 7 days  Age: 35 – 78 years  Female: 25 – 76 %  APACHE II: 4 – 39  SAPS II: 20 – 70  Duration of mechanical ventilation: 2 – 14 days  Failure of extubation/Weaning: 6 – 80%   1. **Diaphragm end-expiratory thickness (TDIee)**   Patients over 16 years of age, on mechanical ventilation for at least 24 to 72 hours  Age: 42 – 78 years  Female: 25 – 76 %  APACHE II: 20 – 39  SAPS II: 20 – 55  Duration of mechanical ventilation: 4 – 14 days  Failure of extubation/Weaning: 9 – 74%   1. **Diaphragm end-inspiratory thickness (TDIee)**   Patients over 18 years of age, on mechanical ventilation for at least 24 hours up 30 days  Age: 59 – 78 years  Female: 26 – 50 %  APACHE II: 20 – 38,5  SAPS II: 34 – 55  Duration of mechanical ventilation: 4 – 14 days  Failure of extubation/Weaning: 9 – 74% | 1. **Maximal inspiratory pressure** with cut-off > - 16 to -50 cmH2O, before, during or after spontaneous ventilation test with T-piece, CPAP or PSV (PS 5 - 8 cmH2O and PEEP 0 - 5 cmH2O). 2. **Diaphragm excursion** with cut-off point < 9.1 – 60 mm, before, during or after spontaneous ventilation test with T-piece, CPAP or PSV (PS 5 – 10 cmH2O, PEEP 0 – 5 cmH2O). 3. **Diaphragm thickening fraction** with cut-off point < 13.5 – 50% before, during or after spontaneous ventilation test with T-piece, CPAP or PSV (PS 5 – 10 cmH2O, PEEP 0 – 10 cmH2O). 4. **Diaphragm end-expiratory thickness** with cut-off point < 1,7 – 15,5 mm during spontaneous ventilation test with T-piece or PSV (PS 6 – 10 cmH2O, PEEP 0 – 5 cmH2O) 5. **Diaphragm end-inspiratory thickness** with cut-off point < 2.6 - 21 mm during spontaneous ventilation test with T-piece or PSV (PS 6 – 10 cmH2O, PEEP 0 – 5) | 1. **Maximal inspiratory pressure**   **Rein**  Reintubation, use of non-invasive ventilation, failure of SBT, or death within 24 hours up to 21 days after extubation.   1. **Diaphragm excursion**   Reintubation, use of non-invasive ventilation, failure of SBT, need for tracheostomy, or death within 48 hours up to 21 days after extubation.   1. **Diaphragm thickening fraction**   Reintubation, use of non-invasive ventilation, failure of SBT, need for tracheostomy, or death within 48 hours up to 7 days after extubation.   1. **Diaphragm end-expiratory thickness**   Reintubation, use of non-invasive ventilation, failure of SBT, need for tracheostomy, or death within 48 hours up to 7 days after extubation.   1. **Diaphragm end-inspiratory thickness**   Reintubation, use of non-invasive ventilation, failure of SBT, or death within 48 hours up to 7 days after extubation. | Diagnostic accuracy studies | Medline (via Pubmed), EMBASE, Web of Science, Cochrane Library and CINAHL data- bases |
| Llamas-Alvarez  2017 | Assess the accuracy of lung and diaphragm ultrasound for predicting weaning outcomes in critically ill adults. | Patients over 18 years of age, on mechanical ventilation for at least 24 – 48 hours  Age: 50 – 63 years  Female: 37 – 44 %  APACHE II: 13  SAPS II: 48  Duration of mechanical ventilation: 4 – 7 days  Failure of extubation/Weaning: 18 – 44% | Lung ultrasound score with cut-off > 7 - 19, performed before, during or after a spontaneous ventilation test with T piece or PSV (PS ≤ 8 cmH2O) | Reintubation, use of non-invasive ventilation, failure of SBT, or death within 48 hours after extubation. | Diagnostic accuracy studies | MEDLINE, the Cochrane Library, Web of Science, Scopus, LILACS, Teseo, Tesis Doctorales en Red, and OpenGrey |
| Chenxia Wu 2023 | Assess the efficacy of diagnostic tests that use ScvO2 for the detection of  extubation failure. | Patients over 18 years of age, on mechanical ventilation for at least 48 hours  Age: 54 – 68 years  Female: 11 – 47 %  APACHE II: 16 - 21  SAPS II: 60  Duration of mechanical ventilation: 4 – 8 days  Failure of extubation/Weaning: 22 – 42% | Δ ScvO2 with cut-off > 38%, performed during spontaneous ventilation test with T piece or PSV (PS ≤ 7 cmH2O) | Reintubation, use of non-invasive ventilation within 48 hours after extubation. | Diagnostic accuracy studies | PubMed, Embase via OVID, and Cochrane Library databases |
| Deschamps 2020 | Evaluate the value of BNP measurement with a SBT as a biomarker to predict liberation from MV among critically ill ICU patients | **ΔBNP or ΔBNP%:**  Patients on mechanical ventilation  Age: 48 – 79 years  Female: 34 – 62%  APACHE II: 18 – 23  Duration of mechanical ventilation: 5 – 10 days  Failure of extubation/Weaning: 15 – 47%  **ΔBNP%:**  Patients on mechanical ventilation  Age: 48 – 8 0years  Female: 34 – 57 %  APACHE II: 18 - 23  Duration of mechanical ventilation: 5 – 10 days  Failure of extubation/Weaning: 20 – 47%  **ΔBNP:**  Patients on mechanical ventilation for at least 48 hours  Age: 62 years  Female: 62 %  APACHE II: 18  SAPS II: 44  Duration of mechanical ventilation: 8 days  Failure of extubation/Weaning: 15 – 41% | **ΔBNP or ΔBNP%:**  ΔBNP**%** with cut-off of 13,4 - 20%, ΔBNP with cut-off of 48 – 80 ng/L or ΔNT-proBNP of 21 ng/L, measured during and 2 h after SBT with T-piece or PSV (PS 8 cmH2O).  **ΔBNP%:**  Δ BNP**%** with cut-off of 13,4 - 20% measured during and 2 h after SBT with T-piece or PSV (PS 8 cmH2O).  **ΔBNP:**  Δ BNP with cut-off of 48 – 80 ng/L ΔNT-proBNP of 21 ng/L, measured during and 2 h after SBT with T-piece or PSV. | **ΔBNP or ΔBNP%:**  Reintubation, use of non-invasive ventilation or failure of SBT within 48 hours after extubation.  **ΔBNP%:**  Reintubation, use of non-invasive ventilation or failure of SBT within 48 hours after extubation.  **ΔBNP:**  Reintubation, use of non-invasive ventilation or failure of SBT within 48 hours after extubation. | Diagnostic accuracy studies | Ovid MEDLINE, Ovid EMBASE, Wiley Cochrane Library, Cochrane Database of Systematic Reviews (CDSR), the Cochrane Central Register of Controlled Trials (CENTRAL), and Web of Science Core Collection via Clarivate Analytics |
| Jun Duan 2021 | Assess the efficacy of diagnostic tests that use cough strength for the early detection of extubation failure. | **Cough peak flow (CPF)**  Patients over 18 years of age, on mechanical ventilation for at least 24 hours up 7 days  Age: 39 - 71 years  Female: 22 – 56 %  APACHE II: 8 – 24  SAPS II: 39 – 48  Duration of mechanical ventilation: 3 – 14 days  Failure of extubation/Weaning: 5 – 41%  **Semiquantitative cough strength score (SCSS)**  Patients over 18 years of age, on mechanical ventilation for at least 24 hours up 21 days  Age: 36 – 77 years  Female: 8 – 53 %  APACHE II: 15 – 25  SAPS II: 21 – 48  Duration of mechanical ventilation: 2 – 25 days  Failure of extubation/Weaning: 7 – 87% | **CPF**  Cough Peak Flow with cut-off ≤ 29,35 - 71,15 L/min, measured before, during or after SBT with T-piece, CPAP or PSV (PS 5 – 8 cmH2O, PEEP 0 – 8 cmH2O)  **SCSS**  Semiquantitative cough strength score measured with SCSS or WCT, classified as negative, weak, ineffective or grado 0 – 2, evaluated before, during or after SBT  with T-piece, CPAP or PSV (PS 5 – 10 cmH2O, PEEP 0 – 5 cmH2O) | **CPF**  Reintubation, use of non-invasive ventilation within 48 hours up to 7 days after extubation.  **SCSS**  Reintubation, use of non-invasive ventilation within 48 hours up to 7 days after extubation. | Diagnostic accuracy studies | PubMed, Web of Science, the Cochrane library, and some Chinese databases (CBM, Wanfang Data, and CNKI) |
| Xie 2025 | Assess the validity of DTF-RSBI as predictors of successful weaning from mechanical ventilation | Patients over 18 years of age, on mechanical ventilation for at least 72 hours  Age: 18 – 82 years  Female: 22 – 34 %  APACHE II: 15 - 20  ISS 42 - 75  Duration of mechanical ventilation: 3 – 14 days  Failure of extubation weaning: 15 – 38% | The patients were examined in either the supine or semi-recumbent position using a linear, high-frequency probe (6–15 MHz) positioned at the mid-clavicular line, anterior to the costal margin, or at the anterior axillary line in the last intercostal space.  Measurements were taken at the end of three inspiratory and three expiratory phases across different respiratory cycles. Diaphragm thickening fraction (DTF) was calculated using the formula: (Thickness at end inspiration—Thickness at end- expiration) divided by Thickness at end-expiration, multiplied by 100 (right and left sides examinate). | Ability to maintain spontaneous breathing within 48 h after extubation without requiring invasive or noninvasive ventilator support or performance of tracheostomy. | Diagnostic accuracy studies | PubMed, Embase, Web of Science, and Cochrane library, and references lists of eligible articles |

**Table 2: Characteristics of Included Reviews: Outcomes**

| **REVIEW** | **INTERVENTION** | **# STUDIES** | **PARTICIPANT DETAILS** | | | **SENSITIVITY** | **SPECIFICITY** |
| --- | --- | --- | --- | --- | --- | --- | --- |
|  |  |  | **N** | **AGE** | **ICU PATHOLOGY** |  |  |
| Sato  2021 | **P0.1**  P0.1 with cut-off point between > 2.3 – 5,5 cmH2O, performed before, during or after spontaneous ventilation test with PS 5-7 cmH2O, T-piece or Automatic tube compensation. | 12 | 1087 | 52 – 70 | COPD (6 – 64%)  Postoperative (15 – 100%)  Pneumonia (12 – 28%)  Neurological condition (3 – 40%)  Sepsis (8 – 22%)  Cardiovascular condition (5 – 17%) | 0.86  (0.72-0.94) | 0.58  (0.37 – 0.76) |
| Kuriyama  2020 | **CUFF LEAK TEST**  1.Quantitative tests: tidal volume variation from < 1325 – 50 ml or 15,5 -57%  2.Qualitative tests: presence or absence of leak around the tube.  Performed during assist-control mechanical ventilation (Vt 6 – 12 ml/Kg, RR 20 bpm) or spontaneous mode (T-piece, PSV or CPAP) | 13 | 2147 | 56 – 67 | Postoperative condition (6 – 100%)  Trauma (48 – 100%)  COPD (6 – 84%)  Sepsis (15 – 39%)  Pneumonia (16 – 47%) | 0.66  (0.46–0.81) | 0.88  (0.83– 0.92) |
| Jia  2024 | **RSBI**  RSBI with cut-off point between > 34,5 - 43.5 - 130 respirations/min/L, performed during or after a spontaneous ventilation test with T-Piece, CPAP or PSV (PS 5 -10 cmH2O, PEEP 0 – 5 cmH2O) | 79 | 13161 | 35 – 79 | Acute respiratory failure (5 – 63%)  Postoperative condition (5 – 100%)  Neurological condition (1 – 100%)  COPD (6 – 100%)  Cardiovascular condition (5 – 16,5%) Pneumonia (7 – 100%) | 0.6  (0.59 – 0.61) | 0.68  (0.66 – 0.7) |
| Sang  2021 | **(D-RSBI)**  D-RSBI with cut-off point between > 1.13 – 1.9, performed before or during spontaneous ventilation test with T-Piece, PSV (PS 5 – 8 cmH2O, PEEP ≤ 5 cmH2O) | 9 | 564 | 36 – 76 | COPD (21 – 100%)  Sepsis (9 – 57%)  Pneumonia (8 – 52%)  Neurological condition (3 – 59%)  ARDS (5 – 55%)  Cardiovascular condition (6 – 11%) | 0.84  (0.76 – 0.9) | 0.87  (0.79 – 0.92) |
| Poddighe  2024 | 1. **Maximal inspiratory pressure (MIP)** with cut-off > - 16 to -50 cmH2O, before, during or after spontaneous ventilation test with T-piece, CPAP or PSV (PS 5 - 8 cmH2O and PEEP 0 - 5 cmH2O). 2. **Diaphragm excursion (DE)**with cut-off point < 9.1 – 60 mm, before, during or after spontaneous ventilation test with T-piece, CPAP or PSV (PS 5 – 10 cmH2O, PEEP 0 – 5 cmH2O). 3. **Diaphragm thickening fraction (DTF)** with cut-off point < 13.5 – 50% before, during or after spontaneous ventilation test with T-piece, CPAP or PSV (PS 5 – 10 cmH2O, PEEP 0 – 10 cmH2O). 4. **Diaphragm end-expiratory thickness (TDI_ee_)** with cut-off point < 1,7 – 15,5 mm during spontaneous ventilation test with T-piece or PSV (PS 6 – 10 cmH2O, PEEP 0 – 5 cmH2O) 5. **Diaphragm end-inspiratory thickness (TDI_ei_)** with cut-off point < 2.6 - 21 mm during spontaneous ventilation test with T-piece or PSV (PS 6 – 10 cmH2O, PEEP 0 – 5) | **MIP**  18  **DE**  53  **DTF**  48  **TDIee**  11  **TDIei**  8 | **MIP**  1118  **DE**  3656  **DTF**  3518  **TDIee**  644  **TDIei**  467 | **MIP**  43 – 70  **DE**  37 – 84  **DTF**  35 – 78  **TDIee**  42 – 78  **TDIei**  59 – 78 | **MIP**  COPD (6 – 100%)  Postoperative condition (7 – 100%)  Sepsis (2 – 69%)  Pneumonia (8 – 53%)  Cardiovascular condition (8 – 41%)  **DE**  COPD (3 – 100%)  Acute respiratory failure (12 – 100%)  Sepsis (5 – 100%)  Pneumonia (7 – 53%)  Neurological condition (5 – 100%)  **DTF**  Acute respiratory failure (1 – 100%)  COPD (4 – 70%)  Pneumonia (7 – 62%)  Sepsis (3 – 100%)  Neurological condition (5 – 47%)  **TDIee**  COPD (7 – 70%)  Acute respiratory failure (16 – 100%)  Pneumonia (7 – 62%)  Cardiovascular condition (10 – 30%)  **TDIei**  Acute respiratory failure (16 – 100%)  COPD (7 – 70%)  Pneumonia (7 – 62%)  Cardiovascular condition (10 – 30%) | **MIP**  **DE**  **DTF**  **TDIee**  **TDIei** | **MIP**  **DE**  **DTF**  **TDIee**  **TDIei** |
| Llamas-Alvarez 2017 | **Lung ultrasound score (LUS Score)**  Lung ultrasound score with cut-off greater than > 7 - 19, performed before, during or after a spontaneous ventilation test with T piece or PSV (PS ≤ 8 cmH2O) | 5 | 324 | 50 – 63 | Acute respiratory failure (35 – 50%)  Postoperative status (6 – 46%)  Cardiovascular disease (32%)  Trauma (7 – 12%) |  |  |
| Chenxia Wu 2023 | **Δ ScvO2**  Δ ScvO2 with cut-off > 38%, performed during spontaneous ventilation test with T piece or PSV (PS ≤ 7 cmH2O) | 5 | 353 | 54 – 68 | Sepsis (49 – 53%)  COPD (1 – 100%)  Pneumonia (56%)  Postoperative condition (7 – 22%) | 0,83  (0,74 – 0,9) | 0,88  (0,83 – 0,92) |
| Deschamps 2020 | **ΔBNP or ΔBNP%:**  ΔBNP**%** with cut-off of 13,4 - 20%, ΔBNP with cut-off of 48 – 80 ng/L or ΔNT-proBNP of 21 ng/L, measured during and 2 h after SBT with T-piece or PSV (PS 8 cmH2O).  **ΔBNP%:**  Δ BNP**%** of 13,4 - 20% measured during and 2 h after SBT with T-piece or PSV (PS 8 cmH2O).  **ΔBNP:**  Δ BNP of 48 – 80 ng/L ΔNT-proBNP of 21 ng/L, measured during and 2 h after SBT with T-piece or PSV. | **ΔBNP or ΔBNP%:**  5  **ΔBNP%:**  5  **ΔBNP:**  3 | **ΔBNP or ΔBNP%:**  227  **ΔBNP%:**  257  **ΔBNP**  222 | **ΔBNP or ΔBNP%:**  48 - 80  **ΔBNP%:**  48 – 80  **ΔBNP**  62 | **ΔBNP or ΔBNP%:**  Pneumonia (26 – 73%)  COPD (9 – 47%)  Sepsis (22 – 29%)  **ΔBNP%:**  Pneumonia (34 – 73%)  COPD (9 – 47%)  Acute respiratory failure (53%)  **ΔBNP**  Sepsis (29%)  Pneumonia (26%)  Neurological condition (19%) | **ΔBNP or ΔBNP%:**  0.89 (0.83 – 0.93)  **ΔBNP%:**  **ΔBNP** | **ΔBNP or ΔBNP%:**  0.83 (0.73 – 0.9)  **ΔBNP%:**  **ΔBNP** |
| Jun Duan 2021 | **Cough peak flow (CPF)**  Cough Peak Flow with cut-off ≤ 29,35 - 71,15 L/min, measured before, during or after SBT with T-piece, CPAP or PSV (PS 5 – 8 cmH2O, PEEP 0 – 8 cmH2O)  **Semiquantitative cough strength score (SCSS)**  Semiquantitative cough strength score measured with SCSS or WCT, classified as negative, weak, ineffective or grado 0 – 2, evaluated before, during or after SBT  with T-piece, CPAP or PSV (PS 5 – 10 cmH2O, PEEP 0 – 5 cmH2O) | **CPF**  19  **SCSS**  20 | **CPF**  2650  **SCSS**  5543 | **CPF**  39 - 71  **SCSS**  36 - 77 | **CPF**  COPD (2 – 100%)  Pneumonia (5 – 40%)  Neurological condition (4 – 100%)  Postoperative condition (3 – 49%)  **SCSS**  Neurological condition (7 – 100%)  Postoperative condition (3 – 31%)  Acute respiratory failure (23 – 46%)  COPD (5 – 100%) | **CPF**  0,76  (0,72 – 0,8)  **SCSS**  0,53  (0,41 – 0,64) | **CPF**  0,75  (0,69 – 0,82)  **SCSS**  0,83  (0,74 – 0,89) |
| Xie 2025 | **DTF-RSBI**  Cut-off point between 48 – 85 breaths/min/percentage, performed before, during or after spontaneous ventilation test with PS <8 cmH2O-PEEP 0-5 cmH2O. T-piece or CPAP inspiratory pressure 0cmH2=, PEEP 5 cmH20 | **4** | **414** | 35 – 71 | COPD (7 – 20%)  Postoperative (0 – 5%)  Pneumonia (0 – 27%)  Neurological condition (0 – 19%)  Sepsis (0 – 8%)  Cardiovascular condition (0 – 11%)  Trauma (0 – 100%) | 0.85  (0.56-0.96) | 0.81  (0.66 – 0.90) |

**Table B2. Characteristics of Excluded Studies**

| **Author / Year** | **Reason for exclusion** |
| --- | --- |
| Di Costanzo D. 2022 ^[1]^ | This publication does not meet the criteria for a systematic review. |
| Girard TD. 2017 ^[2]^ | This publication does not meet the criteria for a systematic review. |
| Gluck E. 1993 ^[3]^ | This publication does not meet the criteria for a systematic review. |
| Karthika M 2023 ^[4]^ | This publication does not meet the criteria for a systematic review. |
| Karthika M 2016 ^[5]^ | This publication does not meet the criteria for a systematic review. |
| Lombardi F. 2019 ^[6]^ | This publication does not meet the criteria for a systematic review. |
| Meade M. 2021 ^[7]^ | This publication does not meet the criteria for a systematic review. |
| Mowafy S. 2019 ^[8]^ | This publication does not meet the criteria for a systematic review. |
| Nemer S. 2011 ^[9]^ | This publication does not meet the criteria for a systematic review. |
| Le-Neindre A. 2021 ^[10]^ | This systematic review and meta-analysis evaluate the diagnostic accuracy of DTF and DE in predicting weaning failure in critically ill patients. Quality assessment using the ROBIS tool revealed low concern in the eligibility domain, but high concern in the identification and selection domain due to inadequate descriptions of other search sources, including unpublished articles. The data collection, study assessment, and synthesis domains showed low concern, and the ROBIS global assessment aligned with other included meta-analyses. However, this study was not prioritized because it is neither the most recent nor does it include a larger population. |
| Li C. 2018 ^[11]^ | This systematic review and meta-analysis assessed the performance of DTF and DE in predicting reintubation within 48 hours after weaning. The ROBIS tool indicated high concern in the eligibility domain due to language restrictions. The identification and selection of studies domain also received a high concern rating because of the exclusion of conference proceedings and the lack of description regarding other search sources for unpublished papers. The data collection and study assessment domain showed low concern, while the synthesis and findings domain raised high concern due to the use of an inappropriate model for calculating the pooled effect. Overall, the ROBIS global assessment was inferior compared to other included meta-analyses. |
| Mahmoodpoor A. 2022 ^[12]^ | This systematic review and meta-analysis evaluate the diagnostic accuracy of DTF, DE, and RSBI in predicting weaning failure in critically ill patients. The ROBIS tool indicated high concern in the eligibility domain due to the absence of a review protocol. The identification and selection of studies domain also received a high concern rating because of the lack of description regarding other search sources for unpublished studies and the use of a search strategy without a clear PICO component. The data collection and study assessment domain showed low concern, while the synthesis and findings domain raised high concern due to the use of an inappropriate model for calculating the pooled effect. Overall, the ROBIS global assessment was inferior compared to other included meta-analyses. |
| Parada-Gereda H. 2023 ^[13]^ | This systematic review and meta-analysis assessed the diagnostic test accuracy of DTF and DE in predicting reintubation within 48 hours after extubation. The ROBIS tool indicated low concern in the eligibility domain. However, the identification and selection of studies domain raised high concern due to the lack of description regarding other search sources for unpublished studies. The data collection and study assessment domain showed low concern, while the synthesis and findings domain also raised high concern due to the use of an inappropriate model for calculating the pooled effect. The ROBIS global assessment was consistent with findings from other included meta-analyses. Nonetheless, this study was not prioritized because it is neither the most recent nor does it include a larger population |
| Qian Z. 2018 ^[14]^ | This systematic review and meta-analysis assessed the diagnostic test accuracy of DTF and DE as predictors of weaning outcomes. The ROBIS tool indicated high concern in the eligibility domain due to the absence of a review protocol. The identification and selection of studies domain also received a high concern rating because of the lack of description regarding other search sources for unpublished studies. The data collection and study assessment domain showed low concern, while the synthesis and findings domain raised high concern due to the use of an inappropriate model for calculating the pooled effect. Overall, the ROBIS global assessment was inferior compared to other included meta-analyses. |
| Trivedi V. 2022 ^[15]^ | This systematic review and meta-analysis assessed the diagnostic test accuracy of RSBI as predictors of weaning outcomes. The ROBIS tool indicated low concern in the eligibility domain. However, the identification and selection of studies domain raised high concern due to the lack of description regarding other search sources for unpublished studies. The data collection and study assessment domain showed low concern, while the synthesis and findings domain also raised high concern due to the use of an inappropriate model for calculating the pooled effect. The ROBIS global assessment was consistent with findings from other included meta-analyses. Nonetheless, this study was not prioritized because it is neither the most recent nor does it include a larger population. |
| Zhou T. 2021 ^[16]^ | This systematic review and meta-analysis assess the diagnostic accuracy of the cuff-leak test for predicting weaning failure in critically ill patients. Quality assessment using the ROBIS tool indicated high concern in the eligibility domain due to the absence of a predefined protocol. Similarly, the identification and selection of studies domain also raised concerns, primarily because of the lack of information on additional search sources for unpublished studies, an inadequate search strategy, and the absence of a clear PICO component in the search approach. Data collection and study assessment were deemed to have a low risk of bias; however, the synthesis and findings criteria raised high concerns due to the use of an inappropriate method for calculating the pooled effect size. Overall, the ROBIS global assessment was less favorable compared to other included meta-analyses. |
| Fernandes Costa F. 2018 ^[17]^ | This systematic review and meta-analysis focus on prognostic factors for weaning failure. Does not provide information on diagnostic test accuracy. |
| Torrini F. 2021 ^[18]^ | This systematic review and meta-analysis focus on prognostic factors for weaning failure. Does not provide information on diagnostic test accuracy. |
| García-Sánchez A. 2020 ^[19]^ | This systematic review and meta-analysis focus on prognostic factors for weaning failure. Does not provide information on diagnostic test accuracy. |
| Truong D. 2023 ^[20]^ | This systematic review and meta-analysis focus on prognostic factors for weaning failure. Does not provide information on diagnostic test accuracy. |
| Van Haute M. 2022 ^[21]^ | Abstract of a systematic review and meta-analysis examining the diagnostic test accuracy of Vd/Vt. This abstract lacks sufficient information for a comprehensive assessment, particularly due to the absence of a clear distinction between adult and pediatric populations. |
| Ding N. 2019 ^[22]^ | Abstract of a systematic review and meta-analysis examining the diagnostic test accuracy of MIP, Vt, RSBI, and P0.1. This abstract lacks adequate information for a comprehensive assessment. |
| Kuriyama A. 2018 ^[23]^ | Abstract of a systematic review and meta-analysis examining the diagnostic test accuracy of cuff test. This abstract lacks sufficient information for a comprehensive assessment. |
| Parry S. 2020 ^[24]^ | Abstract of a systematic review and meta-analysis examining the diagnostic test accuracy of respiratory musculature. This abstract lacks sufficient information for a comprehensive assessment. |
| Tashiro N. 2023 ^[25]^ | Protocol for a systematic review and meta-analysis. |
| Gao Y. 2023 ^[26]^ | Systematic review and meta-analysis examining the diagnostic test accuracy of LUS, DTF, and DE in pediatric population. |
| Van Haute M. 2022 ^[21]^ | Duplicated. |

1] Di Costanzo D, Mazza M, Esquinas A. Diaphragm ultrasound in weaning from mechanical ventilation: a last step to predict successful extubation? Acute and Critical Care 2022;37. https://doi.org/10.4266/acc.2022.00227.

[2] Girard TD, Alhazzani W, Kress JP, Ouellette DR, Schmidt GA, Truwit JD, et al. An Official American Thoracic Society/American College of Chest Physicians Clinical Practice Guideline: Liberation from mechanical ventilation in critically ill adults rehabilitation protocols, ventilator liberation protocols, and cuff leak tests. Am J Respir Crit Care Med 2017;195. https://doi.org/10.1164/rccm.201610-2075ST.

[3] Gluck E, Eubanks DH, Bone RC. Techniques for weaning a patient from mechanical ventilation; when to begin, what method to use, and how to predict outcome. J Crit Illn 1993;8.

[4] Karthika M, Al Enezi FA, Pillai L V., Arabi YM. Rate of Change of Rapid Shallow Breathing Index and Extubation Outcome in Mechanically Ventilated Patients. Crit Care Res Pract 2023;2023. https://doi.org/10.1155/2023/9141441.

[5] Karthika M, Al Enezi F, Pillai L, Arabi Y. Rapid shallow breathing index. Ann Thorac Med 2016;11:167. https://doi.org/10.4103/1817-1737.176876.

[6] Lombardi FS, Cotoia A, Petta R, Schultz M, Cinnella G, Horn J. Prediction of extubation failure in Intensive Care Unit: Systematic review of parameters investigated. Minerva Anestesiol 2019;85. https://doi.org/10.23736/S0375-9393.18.12627-7.

[7] Meade M, Guyatt G, Cook D, Griffith L, Sinuff T, Kergl C, et al. Predicting Success in Weaning From Mechanical Ventilation. Chest 2001;120:400S-424S. https://doi.org/10.1378/chest.120.6_suppl.400S.

[8] Mowafy SMS, Abdelgalel EF. Diaphragmatic rapid shallow breathing index for predicting weaning outcome from mechanical ventilation: Comparison with traditional rapid shallow breathing index. Egypt J Anaesth 2019;35. https://doi.org/10.1016/j.egja.2018.10.003.

[9] Nemer SN, Barbas CSV. Predictive parameters for weaning from mechanical ventilation. Jornal Brasileiro de Pneumologia 2011;37. https://doi.org/10.1590/S1806-37132011000500016.

[10] Le Neindre A, Philippart F, Luperto M, Wormser J, Morel-Sapene J, Aho SL, et al. Diagnostic accuracy of diaphragm ultrasound to predict weaning outcome: A systematic review and meta-analysis. Int J Nurs Stud 2021;117. https://doi.org/10.1016/j.ijnurstu.2021.103890.

[11] Li C, Li X, Han H, Cui H, Wang G, Wang Z. Diaphragmatic ultrasonography for predicting ventilator weaning. Medicine (United States) 2018;97. https://doi.org/10.1097/MD.0000000000010968.

[12] Mahmoodpoor A, Fouladi S, Ramouz A, Shadvar K, Ostadi Z, Soleimanpour H. Diaphragm ultrasound to predict weaning outcome: systematic review and meta-analysis. Anaesthesiol Intensive Ther 2022;54:164–74. https://doi.org/10.5114/ait.2022.117273.

[13] Parada-Gereda HM, Tibaduiza AL, Rico-Mendoza A, Molano-Franco D, Nieto VH, Arias-Ortiz WA, et al. Effectiveness of diaphragmatic ultrasound as a predictor of successful weaning from mechanical ventilation: a systematic review and meta-analysis. Crit Care 2023;27. https://doi.org/10.1186/s13054-023-04430-9.

[14] Qian Z, Yang M, Li L, Chen Y. Ultrasound assessment of diaphragmatic dysfunction as a predictor of weaning outcome from mechanical ventilation: A systematic review and meta-analysis. BMJ Open 2018;8. https://doi.org/10.1136/bmjopen-2017-021189.

[15] Trivedi V, Chaudhuri D, Jinah R, Piticaru J, Agarwal A, Liu K, et al. The Usefulness of the Rapid Shallow Breathing Index in Predicting Successful Extubation. Chest 2022;161. https://doi.org/10.1016/j.chest.2021.06.030.

[16] Zhou T, Zhang HP, Chen WW, Xiong ZY, Fan T, Fu JJ, et al. Cuff-leak test for predicting postextubation airway complications: A systematic review. J Evid Based Med 2011;4. https://doi.org/10.1111/j.1756-5391.2011.01160.x.

[17] Fernandes COSTA F, Cavalcanti Farias PERAZZO R, Leite NÓBREGA JC. Capacidade preditiva de índices de desmame ventilatório no desfecho da extubação de pacientes adultos ventilados mecanicamente: uma revisão sistemática. ASSOBRAFIR Ciência 2018;9.

[18] Torrini F, Gendreau S, Morel J, Carteaux G, Thille AW, Antonelli M, et al. Prediction of extubation outcome in critically ill patients: a systematic review and meta-analysis. Crit Care 2021;25:391. https://doi.org/10.1186/s13054-021-03802-3.

[19] García-Sánchez A, Barbero E, Pintado B, Pérez A, Velasco D, Rodríguez C, et al. Disfunción diafragmática evaluada por ecografía como predictora del fracaso de la extubación: Revisión sistemática y metanálisis. Open Respiratory Archives 2020;2. https://doi.org/10.1016/j.opresp.2020.09.005.

[20] Truong D, Abo S, Whish-Wilson GA, D’Souza AN, Beach LJ, Mathur S, et al. Methodological and Clinimetric Evaluation of Inspiratory Respiratory Muscle Ultrasound in the Critical Care Setting: A Systematic Review and Meta-Analysis. Crit Care Med 2023;51. https://doi.org/10.1097/CCM.0000000000005739.

[21] Van Haute M, Jimenez K, Kumar S, Libozada Z, Lim P, Llamzon M, et al. Utility of dead-space-to-tidal-volume ratio in predicting extubation failure: a systematic review and meta-analysis 2022.

[22] Ding N, Zhigang Z, Caiyun Z, Li Y, Yuchen W, Biantong J, et al. The four weaning index as predictors of mechanical ventilated patients:a systematic review and diagnostic meta-analysis. Intensive Care Med Exp 2019;7(Suppl 3):446–446.

[23] Kuriyama A, Jackson J. 19: CUFF LEAK TEST TO PREDICT POST- EXTUBATION AIRWAY OBSTRUCTION IN ADULTS: A META-ANALYSIS. Crit Care Med 2018;46. https://doi.org/10.1097/01.ccm.0000528075.47384.a9.

[24] Parry SM, Baldwin C, Granger C, Mayer K, Abo S, Paris M, et al. How is ultrasound imaging being used to assess respiratory musculature? A systematic review, 2020. https://doi.org/10.1183/13993003.congress-2020.2979.

[25] Tashiro N, Hasegawa T, Nishiwaki H, Ikeda T, Noma H, Levack W, et al. Clinical utility of diaphragmatic ultrasonography for mechanical ventilator weaning in adults: A study protocol for systematic review and meta-analysis. Health Sci Rep 2023;6. https://doi.org/10.1002/hsr2.1378.

[26] Gao Y, Yin H, Wang MH, Gao YH. Accuracy of lung and diaphragm ultrasound in predicting infant weaning outcomes: a systematic review and meta-analysis. Front Pediatr 2023;11. https://doi.org/10.3389/fped.2023.1211306.
