# Supplementary material for: Diagnostic accuracy of tests for assessing readiness for liberation from mechanical ventilation in adults: an overview of reviews
Source: J Intensive Care. 2026 Jan 14;14:14. doi: 10.1186/s40560-026-00848-9 (PMC12874667; doi:10.1186/s40560-026-00848-9)
Supplement: Supplementary file 3 — Additional file 3. [file 40560_2026_848_MOESM3_ESM.docx]

**Question**: Should airway occlusion pressure at 100 msec be used to diagnose liberation from mechanical ventilation in adults? (1)

| \| Sensitivity \| 0.86 (95% CI: 0.72 to 0.94) \| \| --- \| --- \| \| Specificity \| 0.58 (95% CI: 0.37 to 0.76) \| |  | \| Prevalences \| 30% \| 16% \| 53% \| \| --- \| --- \| --- \| --- \| |  |
| --- | --- | --- | --- | --- | --- | --- | --- | --- | --- | --- | --- |

| Outcome | № of studies (№ of patients) | Study design | Factors that may decrease certainty of evidence | | | | | Effect per 1,000 patients tested | | | Test accuracy CoE |
| --- | --- | --- | --- | --- | --- | --- | --- | --- | --- | --- | --- |
|  |  |  | Risk of bias | Indirectness | Inconsistency | Imprecision | Publication bias | pre-test probability of 30% | pre-test probability of 16% | pre-test probability of 53% |  |
| **True positives** (patients with liberation from mechanical ventilation) | 12 studies 1088 patients | cross-sectional (cohort type accuracy study) | very serious^a^ | not serious | very serious^b^ | not serious | publication bias strongly suspected^c^ | 258 (216 to 282) | 138 (115 to 150) | 456 (382 to 498) | ⨁◯◯◯ Very low^a,b,c^ |
| **False negatives** (patients incorrectly classified as not having liberation from mechanical ventilation) |  |  |  |  |  |  |  | 42 (18 to 84) | 22 (10 to 45) | 74 (32 to 148) |  |
| **True negatives** (patients without liberation from mechanical ventilation) | 12 studies 1088 patients | cross-sectional (cohort type accuracy study) | very serious^a^ | not serious | very serious^b^ | very serious^d^ | publication bias strongly suspected^c^ | 406 (259 to 532) | 487 (311 to 638) | 273 (174 to 357) | ⨁◯◯◯ Very low^a,b,c,d^ |
| **False positives** (patients incorrectly classified as having liberation from mechanical ventilation) |  |  |  |  |  |  |  | 294 (168 to 441) | 353 (202 to 529) | 197 (113 to 296) |  |

#### Explanations

a. Downgraded by 2 levels due to risk of bias. Some limitations regarding index test, and flow and timing domains.

b. Downgraded by 2 levels due to inconsistency. The ROC plot, and the wide range for the prediction region demonstrated a high degree of inconsistency between studies for estimates of sensitivity and specificity.

c. c. Downgraded by 1 level due to publication bias. Evidence of publication biases, assessed by funnel plot and, therefore, the trim-and-fill method.

d. Downgraded by 2 levels due to imprecision. Wide range for the confidence interval for specificity

**Question**: Should Rapid shallow breathing index be used to diagnose liberation from mechanical ventilation in adults? (2)

| \| Sensitivity \| 0.60 (95% CI: 0.59 to 0.61) \| \| --- \| --- \| \| Specificity \| 0.68 (95% CI: 0.66 to 0.70) \| |  | \| Prevalences \| 29% \| 6% \| 66% \| \| --- \| --- \| --- \| --- \| |  |
| --- | --- | --- | --- | --- | --- | --- | --- | --- | --- | --- | --- |

| Outcome | № of studies (№ of patients) | Study design | Factors that may decrease certainty of evidence | | | | | Effect per 1,000 patients tested | | | Test accuracy CoE |
| --- | --- | --- | --- | --- | --- | --- | --- | --- | --- | --- | --- |
|  |  |  | Risk of bias | Indirectness | Inconsistency | Imprecision | Publication bias | pre-test probability of 29% | pre-test probability of 6% | pre-test probability of 66% |  |
| **True positives** (patients with liberation from mechanical ventilation) | 79 studies 13170 patients | cross-sectional (cohort type accuracy study) | serious^a^ | serious^b^ | very serious^c^ | not serious | publication bias strongly suspected^d^ | 174 (171 to 177) | 36 (35 to 37) | 396 (389 to 403) | ⨁◯◯◯ Very low^a,b,c,d^ |
| **False negatives** (patients incorrectly classified as not having liberation from mechanical ventilation) |  |  |  |  |  |  |  | 116 (113 to 119) | 24 (23 to 25) | 264 (257 to 271) |  |
| **True negatives** (patients without liberation from mechanical ventilation) | 79 studies 13170 patients | cross-sectional (cohort type accuracy study) | serious^a^ | serious^b^ | very serious^c^ | not serious | publication bias strongly suspected^d^ | 483 (469 to 497) | 639 (620 to 658) | 231 (224 to 238) | ⨁◯◯◯ Very low^a,b,c,d^ |
| **False positives** (patients incorrectly classified as having liberation from mechanical ventilation) |  |  |  |  |  |  |  | 227 (213 to 241) | 301 (282 to 320) | 109 (102 to 116) |  |

#### Explanations

a. Downgraded by 1 level due to risk of bias. Some limitations regarding patient selection and index test domains

b. Downgraded by 1 level due to applicability concerns. Some limitations regarding patient selection and index domains

c. Downgraded 2 levels due to inconsistency. Forest plots, the ROC plot, and the wide range for the prediction region demonstrated a high degree of inconsistency between studies for estimates of sensitivity and specificity.

d. Downgraded by 1 level due to publication bias. Deeks’ funnel plot asymmetry test yielded a P-value < 0.05, indicating statistically significant asymmetry

**Question**: Should Maximal Inspiratory Pressure be used to diagnose liberation from mechanical ventilation in adults? (3)

| \| Sensitivity \| 0.60 (95% CI: 0.44 to 0.74) \| \| --- \| --- \| \| Specificity \| 0.78 (95% CI: 0.68 to 0.85) \| |  | \| Prevalences \| 31% \| 7% \| 44% \| \| --- \| --- \| --- \| --- \| |  |
| --- | --- | --- | --- | --- | --- | --- | --- | --- | --- | --- | --- |

| Outcome | № of studies (№ of patients) | Study design | Factors that may decrease certainty of evidence | | | | | Effect per 1,000 patients tested | | | Test accuracy CoE |
| --- | --- | --- | --- | --- | --- | --- | --- | --- | --- | --- | --- |
|  |  |  | Risk of bias | Indirectness | Inconsistency | Imprecision | Publication bias | pre-test probability of 31% | pre-test probability of 7% | pre-test probability of 44% |  |
| **True positives** (patients with liberation from mechanical ventilation) | 18 studies 1107 patients | cross-sectional (cohort type accuracy study) | very serious^a^ | serious^b^ | very serious^c^ | not serious | none | 186 (136 to 229) | 42 (31 to 52) | 264 (194 to 326) | ⨁◯◯◯ Very low^a,b,c^ |
| **False negatives** (patients incorrectly classified as not having liberation from mechanical ventilation) |  |  |  |  |  |  |  | 124 (81 to 174) | 28 (18 to 39) | 176 (114 to 246) |  |
| **True negatives** (patients without liberation from mechanical ventilation) | 18 studies 1107 patients | cross-sectional (cohort type accuracy study) | very serious^a^ | serious^b^ | very serious^c^ | not serious | none | 538 (469 to 586) | 725 (632 to 791) | 437 (381 to 476) | ⨁◯◯◯ Very low^a,b,c^ |
| **False positives** (patients incorrectly classified as having liberation from mechanical ventilation) |  |  |  |  |  |  |  | 152 (104 to 221) | 205 (139 to 298) | 123 (84 to 179) |  |

#### Explanations

a. Downgraded by 2 levels due to risk of bias. Several limitations regarding patient selection, index test, reference standard, and flow and timing domains.

b. Downgraded by 1 level due to applicability concerns. Some limitations regarding index text domain.

c. Downgraded by 2 levels due to inconsistency. Forest plots, the ROC plot, and the wide range for the prediction region demonstrated a high degree of inconsistency between studies for estimates of sensitivity and specificity

**Question**: Should Cuff leak test be used to diagnose liberation from mechanical ventilation in adults? (4)

| \| Sensitivity \| 0.66 (95% CI: 0.46 to 0.81) \| \| --- \| --- \| \| Specificity \| 0.88 (95% CI: 0.83 to 0.92) \| |  | \| Prevalences \| 3% \| 0% \| 10% \| \| --- \| --- \| --- \| --- \| |  |
| --- | --- | --- | --- | --- | --- | --- | --- | --- | --- | --- | --- |

| Outcome | № of studies (№ of patients) | Study design | Factors that may decrease certainty of evidence | | | | | Effect per 1,000 patients tested | | | Test accuracy CoE |
| --- | --- | --- | --- | --- | --- | --- | --- | --- | --- | --- | --- |
|  |  |  | Risk of bias | Indirectness | Inconsistency | Imprecision | Publication bias | pre-test probability of 3% | pre-test probability of 0% | pre-test probability of 10% |  |
| **True positives** (patients with liberation from mechanical ventilation) | 28 studies 4493 patients | cross-sectional (cohort type accuracy study) | very serious^a^ | not serious | serious^b^ | serious^c^ | none | 20 (14 to 24) | 0 (0 to 0) | 66 (46 to 81) | ⨁◯◯◯ Very low^a,b,c^ |
| **False negatives** (patients incorrectly classified as not having liberation from mechanical ventilation) |  |  |  |  |  |  |  | 10 (6 to 16) | 0 (0 to 0) | 34 (19 to 54) |  |
| **True negatives** (patients without liberation from mechanical ventilation) | 28 studies 4493 patients | cross-sectional (cohort type accuracy study) | very serious^a^ | not serious | not serious | not serious | none | 854 (805 to 892) | 880 (830 to 920) | 792 (747 to 828) | ⨁⨁◯◯ Low^a^ |
| **False positives** (patients incorrectly classified as having liberation from mechanical ventilation) |  |  |  |  |  |  |  | 116 (78 to 165) | 120 (80 to 170) | 108 (72 to 153) |  |

#### Explanations

a. Downgraded by 2 levels due to risk of bias. Some limitations regarding index test, patient selection, reference standard, and flow and timing domains.

b. Downgraded by 1 level due to inconsistency. Forest plots, the ROC plot, and the wide range for the prediction region demonstrated a high degree of inconsistency between studies for estimates of sensitivity

c. d. Downgraded by 1 level due to imprecision. Lower bound of the 95% confidence interval fell outside the pre-specified acceptable range

**Question**: Should Cough peak flow be used to diagnose liberation from mechanical ventilation in adults? (5)

| \| Sensitivity \| 0.76 (95% CI: 0.72 to 0.80) \| \| --- \| --- \| \| Specificity \| 0.75 (95% CI: 0.69 to 0.81) \| |  | \| Prevalences \| 18% \| 5% \| 41% \| \| --- \| --- \| --- \| --- \| |  |
| --- | --- | --- | --- | --- | --- | --- | --- | --- | --- | --- | --- |

| Outcome | № of studies (№ of patients) | Study design | Factors that may decrease certainty of evidence | | | | | Effect per 1,000 patients tested | | | Test accuracy CoE |
| --- | --- | --- | --- | --- | --- | --- | --- | --- | --- | --- | --- |
|  |  |  | Risk of bias | Indirectness | Inconsistency | Imprecision | Publication bias | pre-test probability of 18% | pre-test probability of 5% | pre-test probability of 41% |  |
| **True positives** (patients with liberation from mechanical ventilation) | 19 studies 2650 patients | cross-sectional (cohort type accuracy study) | serious^a^ | not serious | serious^b^ | not serious | none | 137 (130 to 144) | 38 (36 to 40) | 312 (295 to 328) | ⨁⨁◯◯ Low^a,b^ |
| **False negatives** (patients incorrectly classified as not having liberation from mechanical ventilation) |  |  |  |  |  |  |  | 43 (36 to 50) | 12 (10 to 14) | 98 (82 to 115) |  |
| **True negatives** (patients without liberation from mechanical ventilation) | 19 studies 2650 patients | cross-sectional (cohort type accuracy study) | serious^a^ | not serious | serious^b^ | not serious | none | 615 (566 to 664) | 712 (656 to 770) | 443 (407 to 478) | ⨁⨁◯◯ Low^a,b^ |
| **False positives** (patients incorrectly classified as having liberation from mechanical ventilation) |  |  |  |  |  |  |  | 205 (156 to 254) | 238 (180 to 294) | 147 (112 to 183) |  |

#### Explanations

a. Downgraded by 1 levels due to risk of bias. Some limitations regarding patient selection, and flow and timing domains.

b. Downgraded by 1 level due to inconsistency. Forest plots, the ROC plot, and the wide range for the prediction region demonstrated a high degree of inconsistency between studies for estimates of sensitivity and specificity

**Question**: Should Semiquantitative cough strength score be used to diagnose liberation from mechanical ventilation in adults? (5)

| \| Sensitivity \| 0.53 (95% CI: 0.41 to 0.64) \| \| --- \| --- \| \| Specificity \| 0.83 (95% CI: 0.74 to 0.89) \| |  | \| Prevalences \| 22% \| 7% \| 87% \| \| --- \| --- \| --- \| --- \| |  |
| --- | --- | --- | --- | --- | --- | --- | --- | --- | --- | --- | --- |

| Outcome | № of studies (№ of patients) | Study design | Factors that may decrease certainty of evidence | | | | | Effect per 1,000 patients tested | | | Test accuracy CoE |
| --- | --- | --- | --- | --- | --- | --- | --- | --- | --- | --- | --- |
|  |  |  | Risk of bias | Indirectness | Inconsistency | Imprecision | Publication bias | pre-test probability of 22% | pre-test probability of 7% | pre-test probability of 87% |  |
| **True positives** (patients with liberation from mechanical ventilation) | 20 studies 5543 patients | cross-sectional (cohort type accuracy study) | serious^a^ | not serious | very serious^b^ | not serious | publication bias strongly suspected^c^ | 117 (90 to 141) | 37 (29 to 45) | 461 (357 to 557) | ⨁◯◯◯ Very low^a,b,c^ |
| **False negatives** (patients incorrectly classified as not having liberation from mechanical ventilation) |  |  |  |  |  |  |  | 103 (79 to 130) | 33 (25 to 41) | 409 (313 to 513) |  |
| **True negatives** (patients without liberation from mechanical ventilation) | 20 studies 5543 patients | cross-sectional (cohort type accuracy study) | serious^a^ | not serious | very serious^b^ | not serious | publication bias strongly suspected^c^ | 647 (577 to 694) | 772 (688 to 828) | 108 (96 to 116) | ⨁◯◯◯ Very low^a,b,c^ |
| **False positives** (patients incorrectly classified as having liberation from mechanical ventilation) |  |  |  |  |  |  |  | 133 (86 to 203) | 158 (102 to 242) | 22 (14 to 34) |  |

#### Explanations

a. Downgraded by 1 level due to risk of bias. Some limitations regarding patient selection, and flow and timing domains.

b. Downgraded by 2 levels due to inconsistency. Forest plots, the ROC plot, and the wide range for the prediction region demonstrated a high degree of inconsistency between studies for estimates of sensitivity and specificity

c. c. Downgraded by 1 level due to publication bias. Deeks’ funnel plot asymmetry test showed statistically significant asymmetry (P = 0.

**Question**: Should Lung ultrasound be used to diagnose liberation from mechanical ventilation in adults? (6)

| \| Sensitivity \| 0.94 (95% CI: 0.59 to 0.99) \| \| --- \| --- \| \| Specificity \| 0.87 (95% CI: 0.62 to 0.97) \| |  | \| Prevalences \| 33% \| 17% \| 54% \| \| --- \| --- \| --- \| --- \| |  |
| --- | --- | --- | --- | --- | --- | --- | --- | --- | --- | --- | --- |

| Outcome | № of studies (№ of patients) | Study design | Factors that may decrease certainty of evidence | | | | | Effect per 1,000 patients tested | | | Test accuracy CoE |
| --- | --- | --- | --- | --- | --- | --- | --- | --- | --- | --- | --- |
|  |  |  | Risk of bias | Indirectness | Inconsistency | Imprecision | Publication bias | pre-test probability of 33% | pre-test probability of 17% | pre-test probability of 54% |  |
| **True positives** (patients with liberation from mechanical ventilation) | 5 studies 315 patients | cross-sectional (cohort type accuracy study) | serious^a^ | not serious | very serious^b^ | serious^c^ | none | 310 (195 to 327) | 160 (100 to 168) | 508 (319 to 535) | ⨁◯◯◯ Very low^a,b,c^ |
| **False negatives** (patients incorrectly classified as not having liberation from mechanical ventilation) |  |  |  |  |  |  |  | 20 (3 to 135) | 10 (2 to 70) | 32 (5 to 221) |  |
| **True negatives** (patients without liberation from mechanical ventilation) | 5 studies 315 patients | cross-sectional (cohort type accuracy study) | serious^a^ | not serious | very serious^b^ | serious^c^ | none | 583 (415 to 650) | 722 (515 to 805) | 400 (285 to 446) | ⨁◯◯◯ Very low^a,b,c^ |
| **False positives** (patients incorrectly classified as having liberation from mechanical ventilation) |  |  |  |  |  |  |  | 87 (20 to 255) | 108 (25 to 315) | 60 (14 to 175) |  |

#### Explanations

a. Downgraded by 1 level due to risk of bias. Some limitations regarding patient selection and reference standard domains.

b. Downgraded by 2 levels due to inconsistency. Forest plots, the ROC plot, and the wide range for the prediction region demonstrated a high degree of inconsistency between studies for estimates of sensitivity and specificity

c. Downgraded by 1 level due to imprecision. The lower bound of the 95% confidence interval fell outside the pre-specified acceptable range.

**Question**: Should Diaphragmatic excursion be used to diagnose liberation from mechanical ventilation in adults? (3)

| \| Sensitivity \| 0.74 (95% CI: 0.68 to 0.80) \| \| --- \| --- \| \| Specificity \| 0.80 (95% CI: 0.75 to 0.84) \| |  | \| Prevalences \| 32% \| 5% \| 80% \| \| --- \| --- \| --- \| --- \| |  |
| --- | --- | --- | --- | --- | --- | --- | --- | --- | --- | --- | --- |

| Outcome | № of studies (№ of patients) | Study design | Factors that may decrease certainty of evidence | | | | | Effect per 1,000 patients tested | | | Test accuracy CoE |
| --- | --- | --- | --- | --- | --- | --- | --- | --- | --- | --- | --- |
|  |  |  | Risk of bias | Indirectness | Inconsistency | Imprecision | Publication bias | pre-test probability of 32% | pre-test probability of 5% | pre-test probability of 80% |  |
| **True positives** (patients with liberation from mechanical ventilation) | 53 studies 3641 patients | cross-sectional (cohort type accuracy study) | very serious^a^ | serious^b^ | very serious^c^ | not serious | publication bias strongly suspected^d^ | 237 (218 to 256) | 37 (34 to 40) | 592 (544 to 640) | ⨁◯◯◯ Very low^a,b,c,d^ |
| **False negatives** (patients incorrectly classified as not having liberation from mechanical ventilation) |  |  |  |  |  |  |  | 83 (64 to 102) | 13 (10 to 16) | 208 (160 to 256) |  |
| **True negatives** (patients without liberation from mechanical ventilation) | 53 studies 3641 patients | cross-sectional (cohort type accuracy study) | very serious^a^ | serious^b^ | very serious^c^ | not serious | publication bias strongly suspected^d^ | 544 (510 to 571) | 760 (712 to 798) | 160 (150 to 168) | ⨁◯◯◯ Very low^a,b,c,d^ |
| **False positives** (patients incorrectly classified as having liberation from mechanical ventilation) |  |  |  |  |  |  |  | 136 (109 to 170) | 190 (152 to 238) | 40 (32 to 50) |  |

#### Explanations

a. Downgraded by 2 levels due to risk of bias. Some limitations regarding patient selection, index test, reference standard, and flow and timing domains.

b. Downgraded by 1 level due to applicability concerns. Some limitations regarding index test domains.

c. Downgraded by 2 levels due to inconsistency. Forest plots, the ROC plot, and the wide range for the prediction region demonstrated a high degree of inconsistency between studies for estimates of sensitivity and specificity

d. Downgraded by 1 level due to publication bias. Deeks’ funnel plot asymmetry test yielded a statistically significant P-value (0.01),

**Question**: Should Diaphragmatic rapid shallow breathing index be used to diagnose liberation from mechanical ventilation in adults? (7)

| \| Sensitivity \| 0.84 (95% CI: 0.76 to 0.90) \| \| --- \| --- \| \| Specificity \| 0.87 (95% CI: 0.79 to 0.92) \| |  | \| Prevalences \| 30% \| 19% \| 39% \| \| --- \| --- \| --- \| --- \| |  |
| --- | --- | --- | --- | --- | --- | --- | --- | --- | --- | --- | --- |

| Outcome | № of studies (№ of patients) | Study design | Factors that may decrease certainty of evidence | | | | | Effect per 1,000 patients tested | | | Test accuracy CoE |
| --- | --- | --- | --- | --- | --- | --- | --- | --- | --- | --- | --- |
|  |  |  | Risk of bias | Indirectness | Inconsistency | Imprecision | Publication bias | pre-test probability of 30% | pre-test probability of 19% | pre-test probability of 39% |  |
| **True positives** (patients with liberation from mechanical ventilation) | 9 studies 568 patients | cross-sectional (cohort type accuracy study) | not serious | not serious^a^ | very serious | not serious | none | 252 (228 to 270) | 160 (144 to 171) | 328 (296 to 351) | ⨁⨁◯◯ Low^a^ |
| **False negatives** (patients incorrectly classified as not having liberation from mechanical ventilation) |  |  |  |  |  |  |  | 48 (30 to 72) | 30 (19 to 46) | 62 (39 to 94) |  |
| **True negatives** (patients without liberation from mechanical ventilation) | 9 studies 568 patients | cross-sectional (cohort type accuracy study) | not serious | not serious^a^ | very serious | not serious | none | 609 (553 to 644) | 705 (640 to 745) | 531 (482 to 561) | ⨁⨁◯◯ Low^a^ |
| **False positives** (patients incorrectly classified as having liberation from mechanical ventilation) |  |  |  |  |  |  |  | 91 (56 to 147) | 105 (65 to 170) | 79 (49 to 128) |  |

a. Downgraded by 2 levels due to inconsistency. Forest plots, and the ROC plot demonstrated a high degree of inconsistency between studies for estimates of sensitivity and specificity

**Question**: Should Diaphragmatic thickening fraction be used to diagnose liberation from mechanical ventilation in adults? (3)

| \| Sensitivity \| 0.73 (95% CI: 0.65 to 0.80) \| \| --- \| --- \| \| Specificity \| 0.84 (95% CI: 0.77 to 0.89) \| |  | \| Prevalences \| 31% \| 6% \| 80% \| \| --- \| --- \| --- \| --- \| |  |
| --- | --- | --- | --- | --- | --- | --- | --- | --- | --- | --- | --- |

| Outcome | № of studies (№ of patients) | Study design | Factors that may decrease certainty of evidence | | | | | Effect per 1,000 patients tested | | | Test accuracy CoE |
| --- | --- | --- | --- | --- | --- | --- | --- | --- | --- | --- | --- |
|  |  |  | Risk of bias | Indirectness | Inconsistency | Imprecision | Publication bias | pre-test probability of 31% | pre-test probability of 6% | pre-test probability of 80% |  |
| **True positives** (patients with liberation from mechanical ventilation) | 48 studies 3471 patients | cross-sectional (cohort type accuracy study) | very serious^a^ | not serious | very serious^b^ | not serious | publication bias strongly suspected^c^ | 226 (202 to 248) | 44 (39 to 48) | 584 (520 to 640) | ⨁◯◯◯ Very low^a,b,c^ |
| **False negatives** (patients incorrectly classified as not having liberation from mechanical ventilation) |  |  |  |  |  |  |  | 84 (62 to 108) | 16 (12 to 21) | 216 (160 to 280) |  |
| **True negatives** (patients without liberation from mechanical ventilation) | 48 studies 3471 patients | cross-sectional (cohort type accuracy study) | very serious^a^ | not serious | very serious^b^ | not serious | publication bias strongly suspected^c^ | 580 (531 to 614) | 790 (724 to 837) | 168 (154 to 178) | ⨁◯◯◯ Very low^a,b,c^ |
| **False positives** (patients incorrectly classified as having liberation from mechanical ventilation) |  |  |  |  |  |  |  | 110 (76 to 159) | 150 (103 to 216) | 32 (22 to 46) |  |

#### Explanations

a. Downgraded by 2 levels due to risk of bias. Some limitations regarding patient selection, index test, reference standard, and flow and timing domains.

b. Downgraded by 2 levels due to inconsistency. Forest plots, the ROC plot, and the wide range for the prediction region demonstrated a high degree of inconsistency between studies for estimates of sensitivity and specificity

c. Downgraded by 1 level due to publication bias. Deeks’ funnel plot asymmetry test yielded a P-value of 0.01, indicating statistically significant asymmetry.

**Question**: Should End-expiratory diaphragm thickness be used to diagnose liberation from mechanical ventilation in adults? (3)

| \| Sensitivity \| 0.69 (95% CI: 0.50 to 0.83) \| \| --- \| --- \| \| Specificity \| 0.59 (95% CI: 0.32 to 0.82) \| |  | \| Prevalences \| 34% \| 9% \| 73% \| \| --- \| --- \| --- \| --- \| |  |
| --- | --- | --- | --- | --- | --- | --- | --- | --- | --- | --- | --- |

| Outcome | № of studies (№ of patients) | Study design | Factors that may decrease certainty of evidence | | | | | Effect per 1,000 patients tested | | | Test accuracy CoE |
| --- | --- | --- | --- | --- | --- | --- | --- | --- | --- | --- | --- |
|  |  |  | Risk of bias | Indirectness | Inconsistency | Imprecision | Publication bias | pre-test probability of 34% | pre-test probability of 9% | pre-test probability of 73% |  |
| **True positives** (patients with liberation from mechanical ventilation) | 11 studies 617 patients | cross-sectional (cohort type accuracy study) | very serious^a^ | not serious | very serious^b^ | serious^c^ | none | 235 (170 to 282) | 62 (45 to 75) | 504 (365 to 606) | ⨁◯◯◯ Very low^a,b,c^ |
| **False negatives** (patients incorrectly classified as not having liberation from mechanical ventilation) |  |  |  |  |  |  |  | 105 (58 to 170) | 28 (15 to 45) | 226 (124 to 365) |  |
| **True negatives** (patients without liberation from mechanical ventilation) | 11 studies 617 patients | cross-sectional (cohort type accuracy study) | very serious^a^ | not serious | very serious^b^ | very serious^d^ | none | 389 (211 to 541) | 537 (291 to 746) | 159 (86 to 221) | ⨁◯◯◯ Very low^a,b,d^ |
| **False positives** (patients incorrectly classified as having liberation from mechanical ventilation) |  |  |  |  |  |  |  | 271 (119 to 449) | 373 (164 to 619) | 111 (49 to 184) |  |

#### Explanations

a. Downgraded by 2 levels due to risk of bias. Some limitations regarding patient selection, index test, reference standard, and flow and timing domains.

b. Downgraded by 2 levels due to inconsistency. Forest plots, the ROC plot, and the wide range for the prediction region demonstrated a high degree of inconsistency between studies for estimates of sensitivity and specificity

c. Downgraded by 1 level due to imprecision. The lower bound of the 95% confidence interval fell outside the pre-specified acceptable range for sensibility.

d. Downgraded by 2 levels due to imprecision. Wide range for the confidence interval for specificity.

**Question**: Should End-inspiratory diaphragm thickness be used to diagnose liberation from mechanical ventilation in adults? (3)

| \| Sensitivity \| 0.89 (95% CI: 0.59 to 0.98) \| \| --- \| --- \| \| Specificity \| 0.40 (95% CI: 0.05 to 0.90) \| |  | \| Prevalences \| 35% \| 9% \| 73% \| \| --- \| --- \| --- \| --- \| |  |
| --- | --- | --- | --- | --- | --- | --- | --- | --- | --- | --- | --- |

| Outcome | № of studies (№ of patients) | Study design | Factors that may decrease certainty of evidence | | | | | Effect per 1,000 patients tested | | | Test accuracy CoE |
| --- | --- | --- | --- | --- | --- | --- | --- | --- | --- | --- | --- |
|  |  |  | Risk of bias | Indirectness | Inconsistency | Imprecision | Publication bias | pre-test probability of 35% | pre-test probability of 9% | pre-test probability of 73% |  |
| **True positives** (patients with liberation from mechanical ventilation) | 8 studies 464 patients | cross-sectional (cohort type accuracy study) | very serious^a^ | not serious | very serious^b^ | serious^c^ | none | 312 (207 to 343) | 80 (53 to 88) | 650 (431 to 715) | ⨁◯◯◯ Very low^a,b,c^ |
| **False negatives** (patients incorrectly classified as not having liberation from mechanical ventilation) |  |  |  |  |  |  |  | 38 (7 to 143) | 10 (2 to 37) | 80 (15 to 299) |  |
| **True negatives** (patients without liberation from mechanical ventilation) | 8 studies 464 patients | cross-sectional (cohort type accuracy study) | very serious^a^ | not serious | very serious^b^ | extremely serious^d^ | none | 260 (33 to 585) | 364 (46 to 819) | 108 (14 to 243) | ⨁◯◯◯ Very low^a,b,d^ |
| **False positives** (patients incorrectly classified as having liberation from mechanical ventilation) |  |  |  |  |  |  |  | 390 (65 to 617) | 546 (91 to 864) | 162 (27 to 256) |  |

#### Explanations

a. Downgraded by 2 levels due to risk of bias. Some limitations regarding patient selection and reference standard domains.

b. Downgraded by 2 levels due to inconsistency. Forest plots, the ROC plot, and the wide range for the prediction region demonstrated a high degree of inconsistency between studies for estimates of sensitivity and specificity

c. Downgraded by 1 level due to imprecision. The lower bound of the 95% confidence interval fell outside the pre-specified acceptable range for sensibility

d. Downgraded by 3 levels due to imprecision. Wide range for the confidence interval for specificity.

**Question**: Should Decrease in venous oxygen saturation be used to diagnose liberation from mechanical ventilation in adults? (8)

| \| Sensitivity \| 0.83 (95% CI: 0.74 to 0.90) \| \| --- \| --- \| \| Specificity \| 0.88 (95% CI: 0.83 to 0.92) \| |  | \| Prevalences \| 28% \| 22% \| 42% \| \| --- \| --- \| --- \| --- \| |  |
| --- | --- | --- | --- | --- | --- | --- | --- | --- | --- | --- | --- |

| Outcome | № of studies (№ of patients) | Study design | Factors that may decrease certainty of evidence | | | | | Effect per 1,000 patients tested | | | Test accuracy CoE |
| --- | --- | --- | --- | --- | --- | --- | --- | --- | --- | --- | --- |
|  |  |  | Risk of bias | Indirectness | Inconsistency | Imprecision | Publication bias | pre-test probability of 28% | pre-test probability of 22% | pre-test probability of 42% |  |
| **True positives** (patients with liberation from mechanical ventilation) | 5 studies 353 patients | cross-sectional (cohort type accuracy study) | serious^a^ | not serious | serious^b^ | not serious | none | 232 (207 to 252) | 183 (163 to 198) | 349 (311 to 378) | ⨁⨁◯◯ Low^a,b^ |
| **False negatives** (patients incorrectly classified as not having liberation from mechanical ventilation) |  |  |  |  |  |  |  | 48 (28 to 73) | 37 (22 to 57) | 71 (42 to 109) |  |
| **True negatives** (patients without liberation from mechanical ventilation) | 5 studies 353 patients | cross-sectional (cohort type accuracy study) | serious^a^ | not serious | serious^b^ | not serious | none | 634 (598 to 662) | 686 (647 to 718) | 510 (481 to 534) | ⨁⨁◯◯ Low^a,b^ |
| **False positives** (patients incorrectly classified as having liberation from mechanical ventilation) |  |  |  |  |  |  |  | 86 (58 to 122) | 94 (62 to 133) | 70 (46 to 99) |  |

#### Explanations

a. Downgraded by 1 level due to risk of bias. Some limitations regarding index test domain.

b. Downgraded by 1 level due to inconsistency. Forest plots, and the ROC plot demonstrated a degree of inconsistency between studies for estimates of sensitivity and specificity

**Question**: Should Brain natriuretic peptide be used to diagnose liberation from mechanical ventilation in adults? (9)

| \| Sensitivity \| 0.88 (95% CI: 0.83 to 0.92) \| \| --- \| --- \| \| Specificity \| 0.82 (95% CI: 0.73 to 0.89) \| |  | \| Prevalences \| 32% \| 20% \| 47% \| \| --- \| --- \| --- \| --- \| |  |
| --- | --- | --- | --- | --- | --- | --- | --- | --- | --- | --- | --- |

| Outcome | № of studies (№ of patients) | Study design | Factors that may decrease certainty of evidence | | | | | Effect per 1,000 patients tested | | | Test accuracy CoE |
| --- | --- | --- | --- | --- | --- | --- | --- | --- | --- | --- | --- |
|  |  |  | Risk of bias | Indirectness | Inconsistency | Imprecision | Publication bias | pre-test probability of 32% | pre-test probability of 20% | pre-test probability of 47% |  |
| **True positives** (patients with liberation from mechanical ventilation) | 5 studies 257 patients | cross-sectional (cohort type accuracy study) | very serious^a^ | not serious | not serious | not serious | none | 282 (266 to 294) | 176 (166 to 184) | 414 (390 to 432) | ⨁⨁◯◯ Low^a^ |
| **False negatives** (patients incorrectly classified as not having liberation from mechanical ventilation) |  |  |  |  |  |  |  | 38 (26 to 54) | 24 (16 to 34) | 56 (38 to 80) |  |
| **True negatives** (patients without liberation from mechanical ventilation) | 5 studies 257 patients | cross-sectional (cohort type accuracy study) | very serious^a^ | not serious | not serious | not serious | none | 558 (496 to 605) | 656 (584 to 712) | 435 (387 to 472) | ⨁⨁◯◯ Low^a^ |
| **False positives** (patients incorrectly classified as having liberation from mechanical ventilation) |  |  |  |  |  |  |  | 122 (75 to 184) | 144 (88 to 216) | 95 (58 to 143) |  |

#### Explanations

a. Downgraded by 2 levels due to risk of bias. Some limitations regarding patient selection, index test, and reference standard domains.

**Question**: Should Diaphragmatic thickening fraction rapid shallow breathing index be used to diagnose liberation from mechanical ventilation in adults? (10)

| \| Sensitivity \| 0.85 (95% CI: 0.79 to 0.98) \| \| --- \| --- \| \| Specificity \| 0.65 (95% CI: 0.28 to 1.0) \| |  | \| Prevalences \| 33% \| 15% \| 38% \| \| --- \| --- \| --- \| --- \| |  |
| --- | --- | --- | --- | --- | --- | --- | --- | --- | --- | --- | --- |

| Outcome | № of studies (№ of patients) | Study design | Factors that may decrease certainty of evidence | | | | | Effect per 1,000 patients tested | | | Test accuracy CoE |
| --- | --- | --- | --- | --- | --- | --- | --- | --- | --- | --- | --- |
|  |  |  | Risk of bias | Indirectness | Inconsistency | Imprecision | Publication bias | pre-test probability of 33% | pre-test probability of 15% | pre-test probability of 38% |  |
| **True positives** (patients with liberation from mechanical ventilation) | 4 studies 414 patients | cross-sectional (cohort type accuracy study) | not serious | not serious^a^ | very serious | not serious | none | 281 (261 to 323) | 128 (119 to 147) | 323 (300 to 372) | ⨁⨁◯◯ Low^a^ |
| **False negatives** (patients incorrectly classified as not having liberation from mechanical ventilation) |  |  |  |  |  |  |  | 49 (7 to 69) | 22 (3 to 31) | 57 (8 to 80) |  |
| **True negatives** (patients without liberation from mechanical ventilation) | 4 studies 414 patients | cross-sectional (cohort type accuracy study) | not serious | not serious^a^ | very serious | not serious | none | 435 (188 to 670) | 553 (238 to 850) | 403 (174 to 620) | ⨁⨁◯◯ Low^a^ |
| **False positives** (patients incorrectly classified as having liberation from mechanical ventilation) |  |  |  |  |  |  |  | 235 (0 to 482) | 297 (0 to 612) | 217 (0 to 446) |  |

a. Downgraded by 2 levels due to inconsistency. Forest plots, and the ROC plot demonstrated a high degree of inconsistency between studies for estimates of sensitivity and specificity

**References**

1. Sato R, Hasegawa D, Hamahata NT, Narala S, Nishida K, Takahashi K, et al. The predictive value of airway occlusion pressure at 100 msec (P0.1) on successful weaning from mechanical ventilation: A systematic review and meta-analysis. J Crit Care [Internet]. 2021 Jun 1 [cited 2024 Sep 24];63:124–32. Available from: <https://pubmed.ncbi.nlm.nih.gov/33012587/>
2. Jia D, Wang H, Wang Q, Li W, Lan X, Zhou H, et al. Rapid shallow breathing index predicting extubation outcomes: A systematic review and meta-analysis. Intensive Crit Care Nurs [Internet]. 2024 Feb 1 [cited 2024 Sep 24];80. Available from: https://pubmed.ncbi.nlm.nih.gov/37783181/
3. Poddighe D, Van Hollebeke M, Choudhary YQ, Campos DR, Schaeffer MR, Verbakel JY, et al. Accuracy of respiratory muscle assessments to predict weaning outcomes: a systematic review and comparative meta-analysis. Crit Care [Internet]. 2024 Dec 1 [cited 2024 Sep 24];28(1). Available from: <https://pubmed.ncbi.nlm.nih.gov/38454487/>
4. Kuriyama A, Jackson JL, Kamei J. Performance of the cuff leak test in adults in predicting post-extubation airway complications: a systematic review and meta-analysis. Crit Care [Internet]. 2020 Dec 1 [cited 2024 Sep 24];24(1). Available from: <https://pubmed.ncbi.nlm.nih.gov/33160405/>
5. Duan J, Zhang X, Song J. Predictive power of extubation failure diagnosed by cough strength: a systematic review and meta-analysis. Crit Care [Internet]. 2021 Dec 1 [cited 2024 Sep 24];25(1):1–12. Available from: https://ccforum.biomedcentral.com/articles/10.1186/s13054-021-03781-5
6. Llamas-Álvarez AM, Tenza-Lozano EM, Latour-Pérez J. Diaphragm and Lung Ultrasound to Predict Weaning Outcome: Systematic Review and Meta-Analysis. Chest [Internet]. 2017 Dec 1 [cited 2024 Sep 24];152(6):1140–50. Available from: https://pubmed.ncbi.nlm.nih.gov/28864053/
7. Sang LL, Teng WY, Yang J, Cao LZ. Predictive value of diaphragmatic rapid shallow breathing index in mechanical ventilation weaning: A systematic review and meta-analysis. Signa Vitae. 2021 Jul 1;17(4):34–41.
8. Wu C, Hu L, Shen Q, Xu H, Huang H. Predictive value of extubation failure by decrease in central venous oxygen saturation: A systematic review and meta-analysis. Heliyon [Internet]. 2023 Jul 1 [cited 2024 Sep 24];9(7). Available from: <https://pubmed.ncbi.nlm.nih.gov/37519770/>
9. Deschamps J, Andersen SK, Webber J, Featherstone R, Sebastianski M, Vandermeer B, et al. Brain natriuretic peptide to predict successful liberation from mechanical ventilation in critically ill patients: a systematic review and meta-analysis. Crit Care [Internet]. 2020 May 11 [cited 2024 Sep 24];24(1). Available from: <https://pubmed.ncbi.nlm.nih.gov/32393393/>
10. Xie D, Xu H, Wang F, Wen W, Dong B. Diagnostic accuracy of rapid shallow breathing index based on diaphragm ultrasound predicting successful weaning from mechanical ventilation: A systematic review and meta-analysis. Intensive Crit Care Nurs. 2025 Oct;90:104038. doi: 10.1016/j.iccn.2025.104038.
