# Supplementary material for: Diagnostic accuracy of tests for assessing readiness for liberation from mechanical ventilation in adults: an overview of reviews
Source: J Intensive Care. 2026 Jan 14;14:14. doi: 10.1186/s40560-026-00848-9 (PMC12874667; doi:10.1186/s40560-026-00848-9)
Supplement: Supplementary file 4 — Additional file 4. [file 40560_2026_848_MOESM4_ESM.docx]

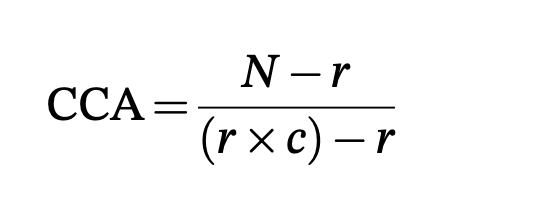


N is the total number of times primary publications appeared in reviews (inclusive of double counting), r is the number of unique primary publications, and c is the number of systematic reviews included in the umbrella review. The formula is accompanied by a classification of the degree of overlap, whereby 0%–5% is considered as “slight overlap”, 6%–10% is considered as “moderate overlap”, 11%–15% is considered as “high overlap”, and >15% is considered as “very high overlap”.

r = 140, N = 245, c = 10, CCA 0.08

| ***N*** | ***Primary publication*** | ***Review***  ***1*** | ***Review***  ***2*** | ***Review 3*** | ***Review 4*** | ***Review***  ***5*** | ***Review***  ***6*** | ***Review 7*** | ***Review***  ***8*** | ***Review***  ***9*** | ***Review***  ***10*** |
| --- | --- | --- | --- | --- | --- | --- | --- | --- | --- | --- | --- |
|  |  | ***Sato 2021*** | ***Kuriyama 2020*** | ***Jia***  ***2024*** | ***Sang 2021*** | ***Poddighe 2024*** | ***Llamas-Álvarez 2017*** | ***Wu 2023*** | ***Deschamps 2020*** | ***Duan***  ***2021*** | ***Xie***  ***2025*** |
| **1** | **Azeredo 2017** | X |  |  |  |  |  |  |  |  |  |
| **2** | **Capdevilla 1995** | X |  |  |  |  |  |  |  |  |  |
| **3** | **Conti 2004** | X |  |  |  | X |  |  |  |  |  |
| **4** | **De Souza 2012** | X |  |  |  | X |  |  |  |  |  |
| **5** | **Fernandez 2004** | X |  |  |  |  |  |  |  |  |  |
| **6** | **Gandia 1992** | X |  |  |  |  |  |  |  |  |  |
| **7** | **Liu 2010** | X |  |  |  |  |  |  |  |  |  |
| **8** | **Montgomery 1987** | X |  |  |  |  |  |  |  |  |  |
| **9** | **Okamoto 1990** | X |  |  |  |  |  |  |  |  |  |
| **10** | **Rivera 1997** | X |  |  |  |  |  |  |  |  |  |
| **11** | **Sassoon 1993** | X |  |  |  | X |  |  |  |  |  |
| **12** | **Vallverdu 1998** | X |  |  |  |  |  |  |  |  |  |
| **13** | **Cheng 2006** |  | X |  |  |  |  |  |  |  |  |
| **14** | **De BasT 2002** |  | X |  |  |  |  |  |  |  |  |
| **15** | **Engoren 1999** |  | X |  |  |  |  |  |  |  |  |
| **16** | **Erginel 2005** |  | X |  |  |  |  |  |  |  |  |
| **17** | **Fisher 1992** |  | X |  |  |  |  |  |  |  |  |
| **18** | **Kriner 2005** |  | X |  |  |  |  |  |  |  |  |
| **19** | **Lee 2007** |  | X |  |  |  |  |  |  |  |  |
| **20** | **Lim 2006** |  | X |  |  |  |  |  |  |  |  |
| **21** | **Maury 2004** |  | X |  |  |  |  |  |  |  |  |
| **22** | **Patel 2015** |  | X |  |  |  |  |  |  |  |  |
| **23** | **Radhi 2012** |  | X |  |  |  |  |  |  |  |  |
| **24** | **Sahbal 2016** |  | X |  |  |  |  |  |  |  |  |
| **25** | **Sandhu 2000** |  | X |  |  |  |  |  |  |  |  |
| **26** | **Abbas 2018** |  |  | **X** |  | **X** |  |  |  | X |  |
| **27** | **Alam 2022** |  |  | **X** |  | **X** |  |  |  |  |  |
| **28** | **Bien Udos 2015** |  |  | X |  |  |  |  |  |  |  |
| **29** | **Boutou 2011** |  |  | X |  |  |  |  |  |  |  |
| **30** | **Bu 2017** |  |  | X |  |  |  |  |  |  |  |
| **31** | **Che 2021** |  |  | X |  |  |  |  |  |  |  |
| **32** | **Cousin 2018** |  |  | X |  |  |  |  |  |  |  |
| **33** | **Danaga 2009** |  |  | X |  |  |  |  |  |  |  |
| **34** | **Delisle 2011** |  |  | X |  |  |  |  |  |  |  |
| **35** | **Dos Reis 2013** |  |  | X |  |  |  |  |  |  |  |
| **36** | **Dres 2012** |  |  | X |  |  |  |  |  |  |  |
| **37** | **Eltrabili 2019** |  |  | **X** |  | **X** |  |  |  |  |  |
| **38** | **Epstein 1995** |  |  | X |  |  |  |  |  |  |  |
| **39** | **Fadaii 2012** |  |  | X |  |  |  |  |  |  |  |
| **40** | **Farghaly 2017** |  |  | **X** |  | **X** |  |  |  |  |  |
| **41** | **Feng 2019** |  |  | **X** | **X** |  |  |  |  |  |  |
| **42** | **Ferrari 2014** |  |  | X |  |  |  |  |  |  |  |
| **43** | **Ghiasi 2019** |  |  | X |  |  |  |  |  |  |  |
| **44** | **Goharani 2019** |  |  | X |  |  |  |  |  |  |  |
| **45** | **Goncalves 2017** |  |  | X |  |  |  |  |  |  |  |
| **46** | **Gong** |  |  | X |  |  |  |  |  |  |  |
| **47** | **Helmy 2021** |  |  | **X** |  | **X** |  |  |  |  |  |
| **48** | **Jin 2019** |  |  | X |  |  |  |  |  |  |  |
| **49** | **Khan 2019** |  |  | X |  |  |  |  |  |  |  |
| **50** | **El Khoury 2010** |  |  | X |  |  |  |  |  |  |  |
| **51** | **Ko 2009** |  |  | X |  |  |  |  |  |  |  |
| **52** | **Krieger 1997** |  |  | X |  |  |  |  |  |  |  |
| **53** | **Kuo 2006** |  |  | X |  |  |  |  |  |  |  |
| **54** | **Kuo 2015** |  |  | X |  |  |  |  |  |  |  |
| **55** | **Lai 2016** |  |  | X |  |  |  |  |  |  |  |
| **56** | **Laz 2019** |  |  | X |  |  |  |  |  |  |  |
| **57** | **Li 2020** |  |  | X |  |  |  |  |  |  |  |
| **58** | **Li 2021** |  |  | **X** |  | **X** |  |  |  |  |  |
| **59** | **Liang 2018** |  |  | X |  |  |  |  |  |  |  |
| **60** | **Lin 2013** |  |  | X |  |  |  |  |  |  |  |
| **61** | **Lin 2019** |  |  | **X** | **X** |  |  |  |  |  |  |
| **62** | **Lin 2021** |  |  | X |  |  |  |  |  |  |  |
| **63** | **Liu 2022** |  |  | X |  |  |  |  |  |  |  |
| **64** | **Luo 2023** |  |  | X |  |  |  |  |  |  |  |
| **65** | **Mahoori 2007** |  |  | X |  |  |  |  |  |  |  |
| **66** | **Mowafy 2019** |  |  | X |  |  |  |  |  |  |  |
| **67** | **Nan 2021** |  |  | X |  |  |  |  |  |  |  |
| **68** | **Okabe 2018** |  |  | **X** |  |  |  |  |  |  |  |
| **69** | **Pirompanich 2018** |  |  | **X** |  | **X** |  |  |  |  |  |
| **70** | **Saiphoklang 2021** |  |  | X |  |  |  |  |  |  |  |
| **71** | **Sanson 2019** |  |  | X |  |  |  |  |  | X |  |
| **72** | **Saravanan 2022** |  |  | **X** |  | **X** |  |  |  |  | X |
| **73** | **Sayed 2019** |  |  | X |  |  |  |  |  |  |  |
| **74** | **Seely 2014** |  |  | X |  |  |  |  |  |  |  |
| **75** | **Segel 2010** |  |  | X |  |  |  |  |  |  |  |
| **76** | **Shamil 2022** |  |  | **X** |  | **X** |  |  |  |  |  |
| **77** | **Song 2022** |  |  | **X** |  | **X** |  |  |  |  | **X** |
| **78** | **Spadaro 2016** |  |  | **X** | **X** | **X** |  |  |  |  |  |
| **79** | **Sun 2017** |  |  | X |  |  |  |  |  |  |  |
| **80** | **Sun 2020** |  |  | X |  |  |  |  |  |  |  |
| **1** | **Takaki 2015** |  |  | X |  |  |  |  |  |  |  |
| **2** | **Tanaka 2016** |  |  | X |  |  |  |  |  |  |  |
| **3** | **Tang 2020** |  |  | X |  |  |  |  |  |  |  |
| **4** | **Tian 2011** |  |  | X |  |  |  |  |  |  |  |
| **5** | **Tian 2022** |  |  | X |  |  |  |  |  |  |  |
| **6** | **Tu 2004** |  |  | X |  |  |  |  |  |  |  |
| **7** | **Uçar 2010** |  |  | X |  |  |  |  |  |  |  |
| **8** | **Wang 2020** |  |  | X |  |  |  |  |  |  |  |
| **9** | **Wang 2021** |  |  | X |  |  |  |  |  |  |  |
| **10** | **Wu 2012** |  |  | X |  |  |  |  |  |  |  |
| **11** | **Wu 2019** |  |  | X |  |  |  |  |  |  |  |
| **12** | **Wu 2021** |  |  | X |  |  |  |  |  |  |  |
| **13** | **Xu 2022** |  |  | **X** |  | **X** |  |  |  |  |  |
| **14** | **Yang 1991** |  |  | X |  |  |  |  |  |  |  |
| **15** | **Yang 1993** |  |  | X |  |  |  |  |  |  |  |
| **16** | **Yang 2022** |  |  | X |  |  |  |  |  |  |  |
| **17** | **Zhang 2010** |  |  | X |  |  |  |  |  |  |  |
| **18** | **Zhang 2013** |  |  | X |  |  |  |  |  |  |  |
| **19** | **Zhang 2014** |  |  | X |  |  |  |  |  |  |  |
| **20** | **Zhang 2018** |  |  | **X** | **X** |  |  |  |  |  |  |
| **21** | **Zhang 2019** |  |  | X |  |  |  |  |  |  |  |
| **22** | **Zhang 2022** |  |  | X |  |  |  |  |  |  |  |
| **23** | **Zhao 2021** |  |  | X |  |  |  |  |  |  |  |
| **24** | **Zhou 2021** |  |  | X |  |  |  |  |  |  |  |
| **25** | **Dou Zhimin 2018** |  |  | X |  |  |  |  |  |  |  |
| **26** | **Abbas 2020** |  |  |  | X |  |  |  |  |  |  |
| **27** | **Dou 2018** |  |  |  | X |  |  |  |  |  |  |
| **28** | **Fan 2018** |  |  |  | X |  |  |  |  |  |  |
| **29** | **Sherif 2018** |  |  |  | X |  |  |  |  |  |  |
| **30** | **Wang 2018** |  |  |  | X |  |  |  |  |  |  |
| **31** | **Abdelhafeez 2019** |  |  |  |  | X |  |  |  |  |  |
| **32** | **Abdelwahed 2019** |  |  |  |  | X |  |  |  |  |  |
| **33** | **Al Tayar 2022** |  |  |  |  | X |  |  |  |  |  |
| **34** | **Ali 2017** |  |  |  |  | X |  |  |  |  |  |
| **35** | **Amara 2022** |  |  |  |  | X |  |  |  |  |  |
| **36** | **Asmita 2022** |  |  |  |  | X |  |  |  |  |  |
| **37** | **Baess 2016** |  |  |  |  | X |  |  |  |  |  |
| **38** | **Banerjee 2018** |  |  |  |  | X |  |  |  |  |  |
| **39** | **Blumhof 2016** |  |  |  |  | X |  |  |  |  |  |
| **40** | **Bruton 2002** |  |  |  |  | X |  |  |  |  |  |
| **41** | **Capdevila 1995** |  |  |  |  | X |  |  |  |  |  |
| **42** | **Carrie 2017** |  |  |  |  | X |  |  |  |  |  |
| **43** | **Cavus 2022** |  |  |  |  | X |  |  |  |  |  |
| **44** | **De Jonghe 2007** |  |  |  |  | X |  |  |  |  |  |
| **45** | **DiNino 2014** |  |  |  |  | X |  |  |  |  |  |
| **46** | **Dres 2021** |  |  |  |  | X |  |  |  |  |  |
| **47** | **Eksombatchai 2023** |  |  |  |  | X |  |  |  |  |  |
| **48** | **Elgazzar 2019** |  |  |  |  | X |  |  |  |  |  |
| **49** | **Elshazly 2020** |  |  |  |  | X |  |  |  |  |  |
| **50** | **Er 2021** |  |  |  |  | X |  |  |  |  |  |
| **51** | **Flevari 2016** |  |  |  |  | X |  |  |  |  |  |
| **52** | **Fossat 2022** |  |  |  |  | X |  |  |  |  |  |
| **53** | **Genty 2022** |  |  |  |  | X |  |  |  |  |  |
| **54** | **Gok 2021** |  |  |  |  | X |  |  |  |  |  |
| **55** | **González-Aguirre 2019** |  |  |  |  | X |  |  |  |  |  |
| **56** | **Haaksma 2021** |  |  |  |  | X |  |  |  |  |  |
| **57** | **Haji 2018** |  |  |  |  | X |  |  |  |  |  |
| **58** | **Hayat 2017** |  |  |  |  | X |  |  |  |  |  |
| **59** | **Hiroli 2023** |  |  |  |  | X |  |  |  |  |  |
| **60** | **Huang 2017** |  |  |  |  | X |  |  |  |  |  |
| **61** | **Huang 2023** |  |  |  |  | X |  |  |  |  |  |
| **62** | **Jiang 2004** |  |  |  |  | X |  |  |  |  |  |
| **63** | **Kaur 2022** |  |  |  |  | X |  |  |  |  |  |
| **64** | **Khan 2018** |  |  |  |  | X |  |  |  |  |  |
| **65** | **Kim 2011** |  |  |  |  | X |  |  |  |  |  |
| **66** | **Kundu 2022** |  |  |  |  | X |  |  |  |  |  |
| **67** | **Lalwani 2022** |  |  |  |  | X |  |  |  |  |  |
| **68** | **Lim 2015** |  |  |  |  | X |  |  |  |  |  |
| **69** | **Luo 2017** |  |  |  |  | X |  |  |  |  |  |
| **70** | **Mariani 2016** |  |  |  |  | X |  |  |  |  |  |
| **71** | **Mawla 2022** |  |  |  |  | X |  |  |  |  |  |
| **72** | **McCool 2020** |  |  |  |  | X |  |  |  |  |  |
| **73** | **Medrinal 2016** |  |  |  |  | X |  |  |  |  |  |
| **74** | **Mohamed 2021** |  |  |  |  | X |  |  |  |  |  |
| **75** | **O´Keefe 2001** |  |  |  |  | X |  |  |  |  |  |
| **76** | **Osman 2017** |  |  |  |  | X | X |  |  |  |  |
| **77** | **Palkar 2018** |  |  |  |  | X |  |  |  |  |  |
| **78** | **Saad 2022** |  |  |  |  | X |  |  |  |  |  |
| **79** | **Saeed 2016** |  |  |  |  | X |  |  |  |  |  |
| **80** | **Saeed 2019** |  |  |  |  | X |  |  |  |  |  |
| **81** | **Samantha 2017** |  |  |  |  | X |  |  |  |  |  |
| **82** | **Soliman 2019** |  |  |  |  | X |  |  |  |  |  |
| **83** | **Spadaro 2021** |  |  |  |  | X |  |  |  |  |  |
| **84** | **Tenza-Lozano 2018** |  |  |  |  | X | X |  |  |  |  |
| **85** | **Thabet 2020** |  |  |  |  | X |  |  |  |  |  |
| **86** | **Threerawit 2018** |  |  |  |  | X |  |  |  |  |  |
| **87** | **Trifi 2021** |  |  |  |  | X |  |  |  |  |  |
| **88** | **Varon-Vega 2021** |  |  |  |  | X |  |  |  |  |  |
| **89** | **Vetrugno 2022** |  |  |  |  | X |  |  |  |  |  |
| **90** | **Vieira 2022** |  |  |  |  | X |  |  |  |  |  |
| **91** | **Vivier 2019** |  |  |  |  | X |  |  |  | X |  |
| **92** | **Yoo 2018** |  |  |  |  | X |  |  |  |  |  |
| **93** | **Zaytoun 2021** |  |  |  |  | X |  |  |  |  |  |
| **94** | **Zhang 2020** |  |  |  |  | X |  |  |  |  |  |
| **95** | **Binet 2014** |  |  |  |  |  | X |  |  |  |  |
| **96** | **Shoaeir 2016** |  |  |  |  |  | X |  |  |  |  |
| **97** | **Soummer 2012** |  |  |  |  |  | X |  |  |  |  |
| **98** | **Ashmawi 2020** |  |  |  |  |  |  | X |  |  |  |
| **99** | **Mallat 2020** |  |  |  |  |  |  | X |  |  |  |
| **100** | **Helmy 2014** |  |  |  |  |  |  | X |  |  |  |
| **101** | **Shalaby 2014** |  |  |  |  |  |  | X |  |  |  |
| **102** | **Teixeira 2010** |  |  |  |  |  |  | X |  |  |  |
| **103** | **Cheng 2015** |  |  |  |  |  |  |  | X |  |  |
| **104** | **Chien 2008** |  |  |  |  |  |  |  | X |  |  |
| **105** | **Farghaly 2015** |  |  |  |  |  |  |  | X |  |  |
| **106** | **Maraghi 2014** |  |  |  |  |  |  |  | X |  |  |
| **107** | **Zapata 2012** |  |  |  |  |  |  |  | X |  |  |
| **108** | **Almeida 2020** |  |  |  |  |  |  |  |  | X |  |
| **109** | **Aziz 2018** |  |  |  |  |  |  |  |  | X |  |
| **110** | **Bai 2017** |  |  |  |  |  |  |  |  | X |  |
| **111** | **Beuret 2009** |  |  |  |  |  |  |  |  | X |  |
| **112** | **Dos 2017** |  |  |  |  |  |  |  |  | X |  |
| **113** | **Duan 2014** |  |  |  |  |  |  |  |  | X |  |
| **114** | **Duan 2015** |  |  |  |  |  |  |  |  | X |  |
| **115** | **Duan 2017** |  |  |  |  |  |  |  |  | X |  |
| **116** | **Elkholy 2021** |  |  |  |  |  |  |  |  | X |  |
| **117** | **Frutos-Vivar 2006** |  |  |  |  |  |  |  |  | X |  |
| **118** | **Gao 2009** |  |  |  |  |  |  |  |  | X |  |
| **119** | **Gobert 2017** |  |  |  |  |  |  |  |  | X |  |
| **120** | **Huang 2013** |  |  |  |  |  |  |  |  | X |  |
| **121** | **Jaber 2019** |  |  |  |  |  |  |  |  | X |  |
| **122** | **Khamiees 2001** |  |  |  |  |  |  |  |  | X |  |
| **123** | **Kutchak 2015** |  |  |  |  |  |  |  |  | X |  |
| **124** | **Liang 2019** |  |  |  |  |  |  |  |  | X |  |
| **125** | **Liu 2014** |  |  |  |  |  |  |  |  | X |  |
| **126** | **Lu 2010** |  |  |  |  |  |  |  |  | X |  |
| **127** | **Ma 2018** |  |  |  |  |  |  |  |  | X |  |
| **128** | **Michetti 2019** |  |  |  |  |  |  |  |  | X |  |
| **129** | **Norisue 2020** |  |  |  |  |  |  |  |  | X |  |
| **130** | **Salam 2004** |  |  |  |  |  |  |  |  | X |  |
| **131** | **Smailes 2013** |  |  |  |  |  |  |  |  | X |  |
| **132** | **Smina 2003** |  |  |  |  |  |  |  |  | X |  |
| **133** | **Su 2010** |  |  |  |  |  |  |  |  | X |  |
| **134** | **Thille 2015** |  |  |  |  |  |  |  |  | X |  |
| **135** | **Thille 2020** |  |  |  |  |  |  |  |  | X |  |
| **136** | **Wang 2009** |  |  |  |  |  |  |  |  | X |  |
| **137** | **Wang 2019** |  |  |  |  |  |  |  |  | X |  |
| **138** | **Xiao 2018** |  |  |  |  |  |  |  |  | X |  |
| **1** | **Sabetian 2024** |  |  |  |  |  |  |  |  |  | X |
| **2** | **Saravanan 2022** |  |  |  |  |  |  |  |  |  | X |
| **3** | **Song 2022** |  |  |  |  |  |  |  |  |  | X |
| **4** | **Long 2020** |  |  |  |  |  |  |  |  |  | X |

**Reference**

1. Kirvalidze M, Abbadi A, Dahlberg L, Sacco LB, Calderón-Larrañaga A, Morin L. Estimating pairwise overlap in umbrella reviews: Considerations for using the corrected covered area (CCA) index methodology. Res Synth Methods. 2023 Sep;14(5):764-767. doi: 10.1002/jrsm.1658.
