# Supplementary material for: Diagnostic accuracy of tests for assessing readiness for liberation from mechanical ventilation in adults: an overview of reviews
Source: J Intensive Care. 2026 Jan 14;14:14. doi: 10.1186/s40560-026-00848-9 (PMC12874667; doi:10.1186/s40560-026-00848-9)
Supplement: Supplementary file 5 — Additional file 5. [file 40560_2026_848_MOESM5_ESM.docx]

| **Index test** | **Variable definition** | | **Sensitivity**  **(CI95%)** | **p Value for subgroup analysis** | **Specificity**  **(CI95%)** | **p Value for subgroup analysis** |  |
| --- | --- | --- | --- | --- | --- | --- | --- |
| P.01^1^ | P0.1 cutoff | Not mentioned | According to review’s information the meta-regression analysis revealed that the effect of cutoff on the predictive value of P0.1 was statistically not significant (p>0.05). | | According to review’s information the meta-regression analysis revealed that the effect of cutoff on the predictive value of P0.1 was statistically not significant (p>0.05). | |  |
|  |  | Not mentioned |  |  |  |  |  |
| Rapid shallow breathing index (RSBI)^2^ | Disease type | COPD | 0.79 (0.76-0.82) | <0.05 | 0.71 (0.66-0.76) | >0.05 |  |
|  |  | Other | 0.86 (0.84-0.89) |  | 0.62 (0.55-0.68) |  |  |
|  | Time for measuring RSBI | Start SBT | 0.75 (0.72, 0.78) | <0.05 | 0.54 (0.48, 0.60) | <0.05 |  |
|  |  | During SBT | 0.78 (0.76, 0.80) |  | 0.66 (0.62, 0.70) |  |  |
|  |  | End SBT | 0.54 (0.52, 0.55) |  | 0.72 (0.69, 0.74) |  |  |
|  | Ventilation method during RSBI | T-tube | 0.79 (0.76, 0.81) | <0.05 | 0.68 (0.64, 0.72) | >0.05 |  |
|  |  | PSV | 0.77 (0.75, 0.79) |  | 0.66 (0.63, 0.70) |  |  |
|  |  | CPAP | 0.60 (0.54, 0.65) |  | 0.81 (0.69, 0.89) |  |  |
|  | RSBI cutoff | ≤80 | 0.55 (0.54, 0.56) | <0.05 | 0.71 (0.68, 0.73) | >0.05 |  |
|  |  | 80 < and ≤ 105 | 0.78 (0.76, 0.80) |  | 0.65 (0.62, 0.68) |  |  |
|  |  | >105 | 0.72 (0.68, 0.75) |  | 0.62 (0.53, 0.71) |  |  |
| Maximal Inspiratory Pressure  (MIP)^3^ | Time for measuring MIP | During MV | According to review’s information the SROC curve comparison with timepoint as potential source of heterogeneity revealed that the effect of variable on the predictive value was statistically not significant (p>0.05). | | According to review’s information the SROC curve comparison with timepoint as potential source of heterogeneity revealed that the effect of variable on the predictive value was statistically not significant (p>0.05). | |  |
|  |  | During SBT |  |  |  |  |  |
|  | MIP cutoff | 16-29 cmH_2_O | According to review’s information the SROC curve comparison with different threshold as potential source of heterogeneity revealed that the effect of variable on the predictive value was statistically not significant (p>0.05). | | According to review’s information the SROC curve comparison with different threshold as potential source of heterogeneity revealed that the effect of variable on the predictive value was statistically not significant (p>0.05). | |  |
|  |  | 30-50 cmH_2_O |  |  |  |  |  |
| Cuff leak test^4^ | Review does not provided information about subgroup analysis. | | | | | |  |
| Cough peak flow^5^ | Subgroup analysis was realized for the variables: voluntary or involuntary CPF; assessment of CPF with an external flowmeter or ventilator; and by different cut-off values. | Not specified | According to the review’s information, the meta-regression analysis revealed that the predictive value of cough peak flow (CPF) does not change significantly by voluntary versus involuntary CPF; CPF measurement with an external flowmeter versus ventilator; or by different cut-off values (p>0.05). | | According to the review’s information, the meta-regression analysis revealed that the predictive value of cough peak flow (CPF) does not change significantly by voluntary versus involuntary CPF; CPF measurement with an external flowmeter versus ventilator; or by different cut-off values (p>0.05). | |  |
| Semiquantitative cough strength score^5^ | Method of measuring the SCSS | Not specified | According to review’s information the meta-regression analysis revealed that the effect of the method used to estimate the SCSC, does not change the predictive value of the index test significant (p>0.05). | | According to review’s information the meta-regression analysis revealed that the effect of the method used to estimate the SCSC, does not change the predictive value of the index test significant (p>0.05). | |  |
| Lung ultrasound score^6^ | Subgroup analysis and bivariate meta-regression were not possible because few studies reported on LUS. | | | | | |  |
| Diaphragmatic excursion (DE)^3^ | | Time for measuring DE | During MV | According to review’s information the SROC curve comparison with timepoint as potential source of heterogeneity revealed that the effect of variable on the predictive value was statistically not significant (p>0.05). | | According to review’s information the SROC curve comparison with timepoint as potential source of heterogeneity revealed that the effect of variable on the predictive value was statistically not significant (p>0.05). | |
|  |  |  | During SBT |  |  |  |  |
|  |  | DE cutoff | 9.1 - 11.9 mm | According to review’s information the SROC curve comparison with the threshold as potential source of heterogeneity revealed that the effect of variable on the predictive value was statistically not significant (p>0.05). | | According to review’s information the SROC curve comparison with the threshold as potential source of heterogeneity revealed that the effect of variable on the predictive value was statistically not significant (p>0.05). | |
|  |  |  | 12.0 - 61 mm |  |  |  |  |
| Diaphragmatic rapid shallow breathing index (D-RSBI)^7^ | | Ventilation method during D-RSBI | PSV | According to review’s information the accuracy comparison according to the ventilation method during D-RSBI as potential source of heterogeneity revealed that the effect of variable on the predictive value was statistically not significant (p>0.05). | | According to review’s information the accuracy comparison according to the ventilation method during D-RSBI as potential source of heterogeneity revealed that the effect of variable on the predictive value was statistically not significant (p>0.05). | |
|  |  |  | T-tube |  |  |  |  |
| Diaphragmatic thickening fraction (DTF)^3^ | | Time for measuring DTF | During MV | According to review’s information the SROC curve comparison with timepoint as potential source of heterogeneity revealed that the effect of variable on the predictive value was statistically not significant (p>0.05). | | According to review’s information the SROC curve comparison with timepoint as potential source of heterogeneity revealed that the effect of variable on the predictive value was statistically not significant (p>0.05). | |
|  |  |  | During SBT |  |  |  |  |
|  |  | DTF cutoff | 13.5 - 29.5 % | According to review’s information the SROC curve comparison with the threshold as potential source of heterogeneity revealed that the effect of variable on the predictive value was statistically not significant (p>0.05). | | According to review’s information the SROC curve comparison with the threshold as potential source of heterogeneity revealed that the effect of variable on the predictive value was statistically not significant (p>0.05). | |
|  |  |  | 30 - 50 % |  |  |  |  |
| End-expiratory diaphragm thickness (EeDT)^3^ | | Time for measuring EeDT | During MV | According to review’s information the SROC curve comparison with timepoint as potential source of heterogeneity revealed that the effect of variable on the predictive value was statistically significant (p<0.05). | | According to review’s information the SROC curve comparison with timepoint as potential source of heterogeneity revealed that the effect of variable on the predictive value was statistically significant (p<0.05). | |
|  |  |  | During SBT |  |  |  |  |
|  |  | EeDT cutoff | 1.7 - 2.89 mm | According to review’s information the SROC curve comparison with the threshold as potential source of heterogeneity revealed that the effect of variable on the predictive value was statistically not significant (p>0.05). | | According to review’s information the SROC curve comparison with the threshold as potential source of heterogeneity revealed that the effect of variable on the predictive value was statistically not significant (p>0.05). | |
|  |  |  | 2.9 – 15.5 mm |  |  |  |  |
| End-inspiratory diaphragm thickness (EiDT)^3^ | | Subgroup analysis and bivariate meta-regression were not possible because few studies reported on EiDT. | | | | | |
| Decrease in venous oxygen saturation (ScvO_2_)^8^ | | Subgroup analysis and bivariate meta-regression were not possible because few studies reported on ScvO_2_. However, systematic review´s authors mentioned a Spearman’s correlation coefficient of 0.1 (p = 0.87), suggestive of no threshold effect. | | | | | |
| Brain natriuretic peptide (Δ BNP%)^9^ | | Subgroup analysis and bivariate meta-regression were not possible because few studies reported on ΔBNP%. | | | | | |
| Diaphragmatic thickening fraction rapid shallow breathing index (DTF-RSBI)^10^ | | Only four studies assessed DTF-RSBI, using varied SBT methods: one with T-tube, one with PSV, one with CPAP, and one unspecified, preventing subgroup analysis by ventilation mode.​ These studies, all from Asian countries, primarily involved male patients without respiratory failure, so subgroup analyses by region, sex, or failure type were infeasible.​ The authors observed that the remaining factors—age, patient positioning, disease type, ultrasound measurement method, and mechanical ventilation duration—also failed to explain heterogeneity. | | | | | |

**References**

1. Sato R, Hasegawa D, Hamahata NT, Narala S, Nishida K, Takahashi K, et al. The predictive value of airway occlusion pressure at 100 msec (P0.1) on successful weaning from mechanical ventilation: A systematic review and meta-analysis. J Crit Care [Internet]. 2021 Jun 1 [cited 2024 Sep 24];63:124–32. Available from: <https://pubmed.ncbi.nlm.nih.gov/33012587/>
2. Jia D, Wang H, Wang Q, Li W, Lan X, Zhou H, et al. Rapid shallow breathing index predicting extubation outcomes: A systematic review and meta-analysis. Intensive Crit Care Nurs [Internet]. 2024 Feb 1 [cited 2024 Sep 24];80. Available from: https://pubmed.ncbi.nlm.nih.gov/37783181/
3. Poddighe D, Van Hollebeke M, Choudhary YQ, Campos DR, Schaeffer MR, Verbakel JY, et al. Accuracy of respiratory muscle assessments to predict weaning outcomes: a systematic review and comparative meta-analysis. Crit Care [Internet]. 2024 Dec 1 [cited 2024 Sep 24];28(1). Available from: <https://pubmed.ncbi.nlm.nih.gov/38454487/>
4. Kuriyama A, Jackson JL, Kamei J. Performance of the cuff leak test in adults in predicting post-extubation airway complications: a systematic review and meta-analysis. Crit Care [Internet]. 2020 Dec 1 [cited 2024 Sep 24];24(1). Available from: <https://pubmed.ncbi.nlm.nih.gov/33160405/>
5. Duan J, Zhang X, Song J. Predictive power of extubation failure diagnosed by cough strength: a systematic review and meta-analysis. Crit Care [Internet]. 2021 Dec 1 [cited 2024 Sep 24];25(1):1–12. Available from: https://ccforum.biomedcentral.com/articles/10.1186/s13054-021-03781-5
6. Llamas-Álvarez AM, Tenza-Lozano EM, Latour-Pérez J. Diaphragm and Lung Ultrasound to Predict Weaning Outcome: Systematic Review and Meta-Analysis. Chest [Internet]. 2017 Dec 1 [cited 2024 Sep 24];152(6):1140–50. Available from: https://pubmed.ncbi.nlm.nih.gov/28864053/
7. Sang LL, Teng WY, Yang J, Cao LZ. Predictive value of diaphragmatic rapid shallow breathing index in mechanical ventilation weaning: A systematic review and meta-analysis. Signa Vitae. 2021 Jul 1;17(4):34–41.
8. Wu C, Hu L, Shen Q, Xu H, Huang H. Predictive value of extubation failure by decrease in central venous oxygen saturation: A systematic review and meta-analysis. Heliyon [Internet]. 2023 Jul 1 [cited 2024 Sep 24];9(7). Available from: <https://pubmed.ncbi.nlm.nih.gov/37519770/>
9. Deschamps J, Andersen SK, Webber J, Featherstone R, Sebastianski M, Vandermeer B, et al. Brain natriuretic peptide to predict successful liberation from mechanical ventilation in critically ill patients: a systematic review and meta-analysis. Crit Care [Internet]. 2020 May 11 [cited 2024 Sep 24];24(1). Available from: <https://pubmed.ncbi.nlm.nih.gov/32393393/>
10. Xie D, Xu H, Wang F, Wen W, Dong B. Diagnostic accuracy of rapid shallow breathing index based on diaphragm ultrasound predicting successful weaning from mechanical ventilation: A systematic review and meta-analysis. Intensive Crit Care Nurs. 2025 Oct;90:104038. doi: 10.1016/j.iccn.2025.104038.
