# Supplementary material for: Diagnostic accuracy of tests for assessing readiness for liberation from mechanical ventilation in adults: an overview of reviews
Source: J Intensive Care. 2026 Jan 14;14:14. doi: 10.1186/s40560-026-00848-9 (PMC12874667; doi:10.1186/s40560-026-00848-9)
Supplement: Supplementary file 6 — Additional file 6. [file 40560_2026_848_MOESM6_ESM.docx]

***Other functional parameters***

One SR (20) assessed other functional parameters used to evaluate readiness for extubation. However, due to the limited number of studies available, statistical combination of the results was impossible. The parameters reported were paraesternal intercostal thickening fraction (two studies, n=205 patients; sensitivity range 0.51 to 0.73 and specificity range 0.81 to 1.00 for a threshold >7.6%); phrenic nerve stimulation (two studies, n=178 patients; sensitivity range 0.76 to 0.81 and specificity range 0.57 to 1.00 for a threshold <8.7cmH2O); maximal expiratory pressure (two studies, n=164 participants; sensitivity range 0.35 to 0.63 and specificity range 0.51 to 0.71 for a threshold >30 cmH2O); diaphragmatic electric activity (two studies, n=64 patients; sensitivity range 0.33 to 0.59 and specificity range 0.83 to 1.00 for a threshold <14.9 μV); thickness of transversus abdominis muscle (one study, n=81 patients; sensitivity 0.63 and specificity range 0.65 for a threshold >2.5 mm); thickness of external oblique muscle (one study, n=81 patients; sensitivity 0.69 and specificity range 0.66 for a threshold >3.1 mm); thickness of internal oblique muscle (one study, n=81 patients; sensitivity 0.69 and specificity range 0.77 for a threshold >4.9 mm); thickness of rectus abdominis muscle (one study, n=81 patients; sensitivity 0.69 and specificity range 0.65 for a threshold <6.3 mm); and transdiaphragmatic pressure (one study, n= 20 participants; sensitivity 1.00 and specificity 0.75 for a threshold >40 cmH2O).

**Reference**

Poddighe D, Van Hollebeke M, Choudhary YQ, Campos DR, Schaeffer MR, Verbakel JY, et al. Accuracy of respiratory muscle assessments to predict weaning outcomes: a systematic review and comparative meta-analysis. Crit Care [Internet]. 2024 Dec 1 [cited 2024 Sep 24];28(1). Available from: https://pubmed.ncbi.nlm.nih.gov/38454487/
